# Modelling integrated antiretroviral treatment and harm reduction services on HIV and overdose among people who inject drugs in Tijuana, Mexico

**DOI:** 10.1002/jia2.25493

**Published:** 2020-06-19

**Authors:** Javier A Cepeda, Annick Bórquez, Christopher Magana, Anh Vo, Claudia Rafful, Gudelia Rangel, María E Medina‐Mora, Steffanie Strathdee, Natasha K Martin

**Affiliations:** ^1^ Division of Infectious Diseases and Global Public Health University of California San Diego San Diego CA USA; ^2^ Faculty of Psychology Universidad Nacional Autonoma de Mexico Mexico City Mexico; ^3^ Center on Global Mental Health Research National Institute on Psychiatry Ramón de la Fuente Muñiz Mexico City Mexico; ^4^ Centre on Drug Policy Evaluation St. Michael’s Hospital Toronto Canada; ^5^ Comisión de Salud Fronteriza México‐Estados Unidos Tijuana Mexico; ^6^ El Colegio de la Frontera Norte Tijuana Mexico; ^7^ Population Health Sciences University of Bristol Bristol United Kingdom

**Keywords:** inject drugs, opioid agonist therapy, HIV, integration, Mexico, overdose, drug treatment

## Abstract

**Introduction:**

The HIV epidemic in Tijuana, Mexico is concentrated in key populations, including people who inject drugs (PWID). However, HIV interventions among PWID are minimal, and federal funding was provided for compulsory abstinence programmes associated with HIV and overdose. Alternatively, opioid agonist therapy reduces overdose, reincarceration, HIV, while improving antiretroviral therapy (ART) outcomes. We assessed potential impact and synergies of scaled‐up integrated ART and opioid agonist therapy, compared to scale‐up of each separately, and potential harms of compulsory abstinence programmes on HIV and fatal overdose among PWID in Tijuana.

**Methods:**

We developed a dynamic model of HIV transmission and overdose among PWID in Tijuana. We simulated scale‐up of opioid agonist therapy from zero to 40% coverage among PWID. We evaluated synergistic benefits of an integrated harm reduction and ART scale‐up strategy (40% opioid agonist therapy coverage and 10‐fold ART recruitment), compared to scale‐up of each intervention alone or no scale‐up of low coverage ART and no harm reduction). We additionally simulated compulsory abstinence programmes (associated with 14% higher risk of receptive syringe sharing and 76% higher odds of overdose) among PWID.

**Results:**

Without intervention, HIV incidence among PWID could increase from 0.72 per 100 person‐years (PY) in 2020 to 0.92 per 100 PY in 2030. Over ten years, opioid agonist therapy scale‐up could avert 31% (95% uncertainty interval (UI): 18%, 46%) and 22% (95% UI: 10%, 28%) new HIV infections and fatal overdoses, respectively, with the majority of HIV impact from the direct effect on HIV transmission due to low ART coverage. Integrating opioid agonist therapy and ART scale‐up provided synergistic benefits, with opioid agonist therapy effects on ART recruitment/retention averting 9% more new infections compared to ART scale‐up alone. The intervention strategy could avert 48% (95% UI: 26%, 68%) of new HIV infections and one‐fifth of fatal overdoses over ten years. Conversely, compulsory abstinence programmes could increase HIV and overdoses.

**Conclusions:**

Integrating ART with opioid agonist therapy could provide synergistic benefits and prevent HIV and overdoses among PWID in Tijuana, whereas compulsory abstinence programmes could cause harm. Policymakers should consider the benefits of integrating harm reduction and HIV services for PWID.

## 1 Introduction

As global funding for HIV prevention and treatment programmes declines, service providers will need to maximize the benefits of their programmes by integrating and identifying potential synergies with other health outcomes affecting those populations [1]. This is critical in resource‐limited settings like Mexico, which has a concentrated HIV epidemic among key populations including people who inject drugs (PWID), female sex workers and men who have sex with men. In Tijuana, Mexico the withdrawal of Global Fund support resulted in decreased likelihood of PWID obtaining clean syringes [2, 3]. Additionally, in 2019, the federal government announced it was suspending funding of civil society organizations responsible for delivering HIV treatment and prevention services [4].

Tijuana, a major city bordering San Diego, California, has one of the highest per‐capita rates of injection drug use in Mexico [5]. HIV prevalence among PWID ranges from 4% to 9% [6], and incidence among female PWID is 2.3 per 100 person‐years, which is among the highest in North America [7]. PWID in Tijuana additionally experience high rates of mortality (4%/year), with approximately one‐quarter of deaths due to drug overdose [8].

Findings from global systematic reviews indicate that opioid agonist therapy, the “gold standard” to treat opioid use disorder [9, 10, 11], is effective in reducing HIV transmission [12], improving antiretroviral therapy (ART) outcomes [13], preventing opioid overdoses [14] and reducing recidivism [15]. Despite these benefits, affordable and quality opioid agonist therapy is limited in Tijuana. In 2009, the Mexican government enacted a public‐health oriented drug law reform which mandated drug treatment for those apprehended under possession thresholds on their third infraction. However, in 2014, some funding from the federal government to address drug use was allocated to compulsory abstinence programmes. This entailed the forced detention of PWID within abstinence‐based drug centres where no evidence‐based drug treatment or medical services to address co‐morbidities (mental health, infectious or chronic disease) are provided [16, 17]. In Mexico, compulsory abstinence programmes have been associated with numerous harms, including increasing odds of non‐fatal overdose by 76% (adjusted odds ratio: 95% CI: 1.05 to 2.96) [18] and risk of receptive syringe sharing (RR = 1.14, 95% CI: 1.00 to 1.30) which could facilitate HIV acquisition [16].

A large evidence base supports the synergistic roles opioid agonist therapy can play in improving PWID health. In addition to reducing opioid overdose risk [14], global systematic reviews/meta‐analyses found that opioid agonist therapy reduced the risk of HIV acquisition by 54% [12] and PWID living with HIV on opioid agonist therapy were 1.68 times more likely to be recruited into HIV care and 23% less likely to drop off of ART [13]. Opioid agonist therapy can reduce reincarceration [15], further reducing HIV [19] and fatal overdose, which is significantly elevated within the first few weeks after release [20].

Mathematical modelling can aid policymakers in making evidence‐based decisions on scaling‐up interventions to enhance public health impact [1, 21]. Our previous modelling of Mexican drug policy reforms found limited HIV impact due to improper implementation. However, if the reform were correctly implemented, then it could have averted over 20% of new HIV infections among PWID in Tijuana by 2030 [16]. Given changing drug policies and HIV funding landscape, our aim was to assess the potential synergies and benefits of an integrated HIV and harm reduction response on HIV and overdose among PWID in Tijuana.

## 2 Methods

### 2.1 Model structure

We extended our previously published dynamic, deterministic model of HIV transmission (sexual‐ and injecting‐related) among PWID in Tijuana [16] to explicitly include fatal overdose and additional opioid agonist therapy effects/synergies on overdose, ART and reincarceration – (Figure S1). Briefly, the model was stratified by sex (male or female), incarceration history (never, in prison, recently incarcerated (past six months), not recently incarcerated), recent police harassment (having syringe confiscated by police or not), opioid agonist therapy or compulsory abstinence programme status (on/off) and HIV stage (susceptible, acute, latent, pre‐AIDS, AIDS) and ART status (see Supplementary material). We modelled injection and sexual transmission as a function of the number of syringe sharing acts, unprotected sex acts, proportion of sexual partners who injected drugs, probability of transmission from risky injection‐ or sexual‐related exposures, HIV prevalence and weighted HIV stage among partners (with greater transmissibility due to higher viraemia if in the acute or pre‐AIDS stage compared to the latent stage) [22, 23]. We assumed ART reduced HIV mortality, sexual HIV transmission and injecting‐related HIV transmission [24]. We assumed proportionate mixing with regards to syringe sharing. Sexual transmission was exclusively heterosexual between PWID and their sexual partners who injected and did not inject drugs.

**Figure 1:**
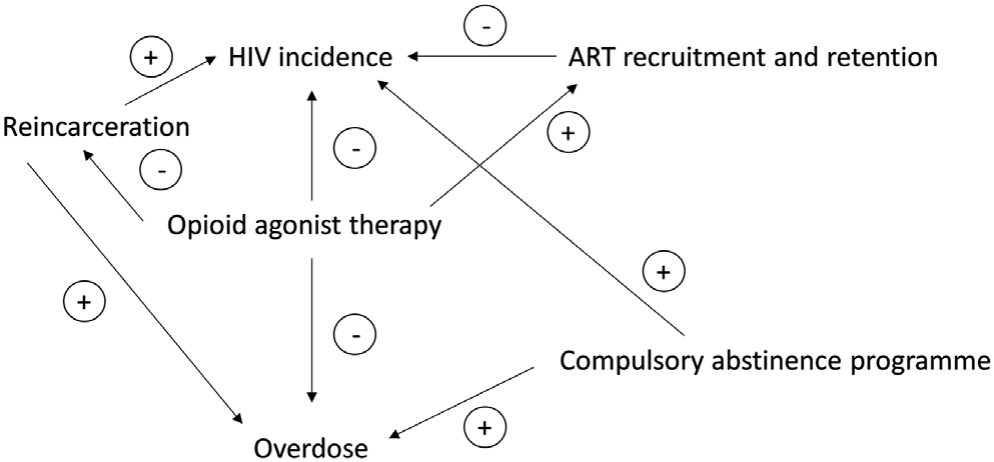
Conceptual model of association between opioid agonist therapy and compulsory abstinence programmes on HIV‐ and drug‐related outcomes. Plus and minus signs correspond to direction of association between exposure (opioid agonist therapy or compulsory abstinence programme) and the outcome (e.g. opioid agonist therapy reduces the risk of overdose (‐) while compulsory abstinence programmes increased the risk (+)).

Figure 1 provides a conceptual overview of how opioid agonist therapy and compulsory abstinence programmes interact with HIV prevention, ART and overdose outcomes in the model. We explicitly incorporated fatal overdose, with elevated risk of overdose within the first four weeks after release from prison [14]. Fatal overdose was reduced by 79% when on opioid agonist therapy, with small elevated risks of fatal overdose upon entering and exiting opioid agonist therapy [14]. We assumed risk of HIV acquisition was reduced while on opioid agonist therapy [12]. Based on a meta‐analysis, recruitment and retention on ART were elevated among people on opioid agonist therapy compared to those not [13]. We also included reduced risk of reincarceration among PWID on opioid agonist therapy [20]. Additionally, ever being exposed to compulsory abstinence programmes increased receptive syringe sharing by 14% [16]. It is unclear whether this elevated risk in syringe sharing occurs during and/or after exposure, or whether this association is causal. Findings from incarcerated settings, where drug use is prohibited, indicate that fewer PWID continue to inject drugs while confined. However, risky injection practices such as syringe sharing occur more frequently due to limited availability of clean syringes [25, 26]. Thus, we implemented the effect of compulsory abstinence programmes exposure in the model by assuming a 14% increase (varying from 0% to 30%) in receptive syringe sharing among PWID while in these programmes. Evidence from Tijuana also indicated that compulsory abstinence programmes were associated with 76% higher odds of recent overdose compared to PWID with no recent exposure [18]. Due to limited data, we assumed that risk of fatal overdose associated with compulsory abstinence programmes would be equivalent to non‐fatal overdose risk.

### 2.2 Model parameterization and calibration

The model was primarily parameterized by demographic and behavioural data from El Cuete IV, a longitudinal cohort of PWID in Tijuana followed from 2011 to 2020 [27]. The El Cuete IV study protocol was approved by the UCSD Human Subjects Protections Program and El Colegio de la Frontera Norte in Tijuana and all participants consented to study procedures. We calibrated to HIV prevalence by sex and incarceration history (in 2005, 2006 and 2011), HIV incidence (in 2014), and ART coverage among HIV‐infected PWID (in 2012). See supplemental material and Borquez *et al* [16] for calibration details. We assumed ART scale‐up began in 2003, varying uniformly to reach 2% to 18% coverage in 2012 [28, 29]) (Figure S2), and no opioid agonist therapy based on low current methadone use (<5% in 2016 to 2018). We sampled 120,000 parameter sets from assigned prior distributions using Latin Hypercube Sampling, calculating the total log‐likelihood as the sum log likelihoods for each of our calibration parameters, and selecting runs producing total log‐likelihoods above the 99^th^ percentile for the final analysis; 201 runs were selected with most projections within the 95% uncertainty intervals (UI) of the data (Figures S3,S4). Key parameters related to ART, opioid agonist therapy, compulsory abstinence programmes and overdose are in Table 1. The remaining model parameters and calibration values are in Tables S1, S2 respectively. Overdose deaths in Mexico are not officially monitored or reported. The overdose rate was therefore based on an epidemiological study using El Cuete IV data which used both confirmed and unconfirmed cause of death information [8]. Given uncertainty and challenges in confirming deaths and inconsistencies in reporting “overdose” or “drug toxicity” as cause of death, we instead used it to generate the overdose mortality rate rather than for calibration.

**Table 1:** Key model input parameters associated with opioid agonist therapy, compulsory abstinence programmes and HIV treatment, prevention and overdose outcomes

Parameter	Symbol	Sampled point estimate and 95% confidence interval	Sampling distribution	Reference/notes
Fatal overdose rate (per year)	μ	0.01 (0.008 to 0.012)	Uniform	[8]
Relative risk of overdose if on opioid agonist therapy (OAT) compared to off opioid agonist therapy	RROD^OAT^	0.21 (0.13 to 0.34)	Lognormal	[14]
Relative risk of overdose if in compulsory abstinence programme compared (CAP) to no compulsory abstinence programme	RROD^CAP^	1.76 (1.05 to 2.96)	Lognormal	[18] Assumed similar to risk of non‐fatal overdose for recent compulsory abstinence programme exposure
Relative risk of fatal overdose within first four weeks after entering opioid agonist therapy compared to being enrolled in opioid agonist therapy programme	RROD^OATin^	1.97 (0.94 to 4.10)	Lognormal	[14]
Relative risk of fatal overdose within first four weeks after exiting opioid agonist therapy compared to being enrolled in opioid agonist therapy programme	RROD^OATout^	2.38 (1.51 to 3.74)	Lognormal	[14]
Relative risk of being recruited on to ART if on opioid agonist therapy compared to off opioid agonist therapy	RR^ARTrecruit^	1.69 (1.32 to 2.15)	Lognormal	[13]
Relative risk of ART discontinuation if on opioid agonist therapy compared to off opioid agonist therapy	RR^ARTdropout^	0.77 (0.63 to 0.95)	Lognormal	[13]
ART discontinuation rate per year	ψ	0.06 (0.02 to 0.10)	Uniform	[30]
Rate of opioid agonist therapy or compulsory abstinence programme cessation (/year)	δ	1 year (varied from 6 months to 2 years in sensitivity analyses		Duration on opioid agonist therapy or compulsory abstinence programmes was assumed to be one year, consistent with the average duration of opioid agonist therapy in low/middle income settings [31]. Average duration in compulsory abstinence programmes in Mexico remains unknown [32] but presumed to average between six months and one year.
Relative risk of recidivism if on opioid agonist therapy compared to off opioid agonist therapy	RR^recid^	0.80 (0.71 to 0.90)	Lognormal	[15]
Relative risk of fatal overdose within first four weeks of release from prison compared to being in opioid agonist therapy programme	RROD^prison^	1.7 (1.3 to 2.2)	Lognormal	[20]
Relative efficacy of ART on parenteral transmission	RR^ARTinj^	0.5 (0.25 to 0.75)	Uniform	[24] Uncertain, varied widely
Relative efficacy of ART on sexual transmission	RR^ARTsex^	0.07 (0.02 to 0.22)	Lognormal	[33]
Relative risk of HIV transmission if on opioid agonist therapy compared to not on opioid agonist therapy	RR^HIVOAT^	0.46 (0.32 to 0.67)	Lognormal	[12]
Relative risk of receptive syringe sharing if in compulsory abstinence programme (CAP) compared to not in compulsory abstinence programme	RR^CAP^	1.14 (1.00 to 1.30)	Lognormal	[16] Assumed similar to risk observed among those ever exposed to compulsory abstinence programme versus not

Other model parameters and calibration data can be found in Tables S1, S2.

### 2.3 Modelled scenarios and outcomes

We assessed the relative benefits of an integrated harm reduction and HIV response, compared to scale‐up strategies which focused on only one aspect, and potential synergistic effects of this integration through ART. As such, we first examined the impact of each component of opioid agonist therapy scale‐up (through its effects on ART recruitment/retention, HIV transmission, and/or incarceration) on HIV incidence, fatal overdose and ART coverage. We then assessed the total and synergistic benefits of combined integrated opioid agonist therapy and ART scale‐up, compared to impact of scale‐up of each intervention alone (opioid agonist therapy or ART) or no scale‐up. Finally, given funding of compulsory abstinence programmes, we explored potential harms of scale‐up of this intervention only.

1. Status quo. No opioid agonist therapy and low ART coverage (ranging from 2% to 18% virally suppressed on ART in 2012).
2. Opioid agonist therapy scale‐up to 40% coverage among PWID from 2020. 40% coverage considered by the World Health Organization to be the threshold for high coverage [35] (Figure S5).
  - Simulating opioid agonist therapy effect on improving ART recruitment/retention only.
  - Simulating opioid agonist therapy direct effect on reducing HIV acquisition only.
  - Simulating full effects: reduced HIV acquisition, increased ART recruitment/retention, reduced reincarceration.
3. ART scale‐up. Recruitment of ART was increased ten‐fold from 2020, reaching a mean of 56% of HIV‐infected PWID on ART by 2030 (similar to current national ART coverage estimates in Mexico [36]).
4. Integrated ART and opioid agonist therapy scale‐up. ART recruitment increased ten‐fold, opioid agonist therapy scaled‐up to 40% among PWID from 2020. We adopted a broad definition of “integrated opioid agonist therapy and HIV services” as it has been implemented either through coordination of services [37] or collocation in one clinical setting [38, 39].
  - Simulating opioid agonist therapy effect on ART recruitment/retention only
  - Simulating full opioid agonist therapy effects: reduced HIV acquisition, increased ART recruitment/retention, reduced reincarceration.
5. Compulsory abstinence programme scale‐up to 40%. Compulsory abstinence programme scaled‐up from zero to 40% coverage among PWID from 2020.

We projected HIV prevalence, HIV incidence, ART coverage and fatal overdose among PWID until 2030. We estimated the proportion of new HIV infections and fatal overdoses averted from 2020 to 2030 between each scenario and the status quo (base case). To assess synergies between opioid agonist therapy and ART, we calculated the difference in the cumulative number of HIV cases from 2020 to 2030 in the integrated scale‐up scenario and the ART scale‐up only scenario.

### 2.4 Sensitivity analyses

We explored impact of uncertainty in intervention parameters on projections of HIV and overdose impact with the integrated OAT and ART scenario compared to the status quo. We conducted several one‐way sensitivity analyses by varying: opioid agonist therapy duration (six months or two years, compared to one year at baseline), opioid agonist therapy effect estimate (“best case scenario” using upper bound values for all effects on ART recruitment retention, HIV transmission, reincarceration and overdose, and a “worst‐case scenario” using the lower bound effect estimates). Due to uncertainty in compulsory abstinence programme effects, we explored how impact of compulsory abstinence programme scale‐up varied with a “best‐case scenario” using the lower bound impact on elevated syringe sharing and overdose, and “worst‐case scenario” using upper bound impact.

## 3 Results

**Figure 2:**
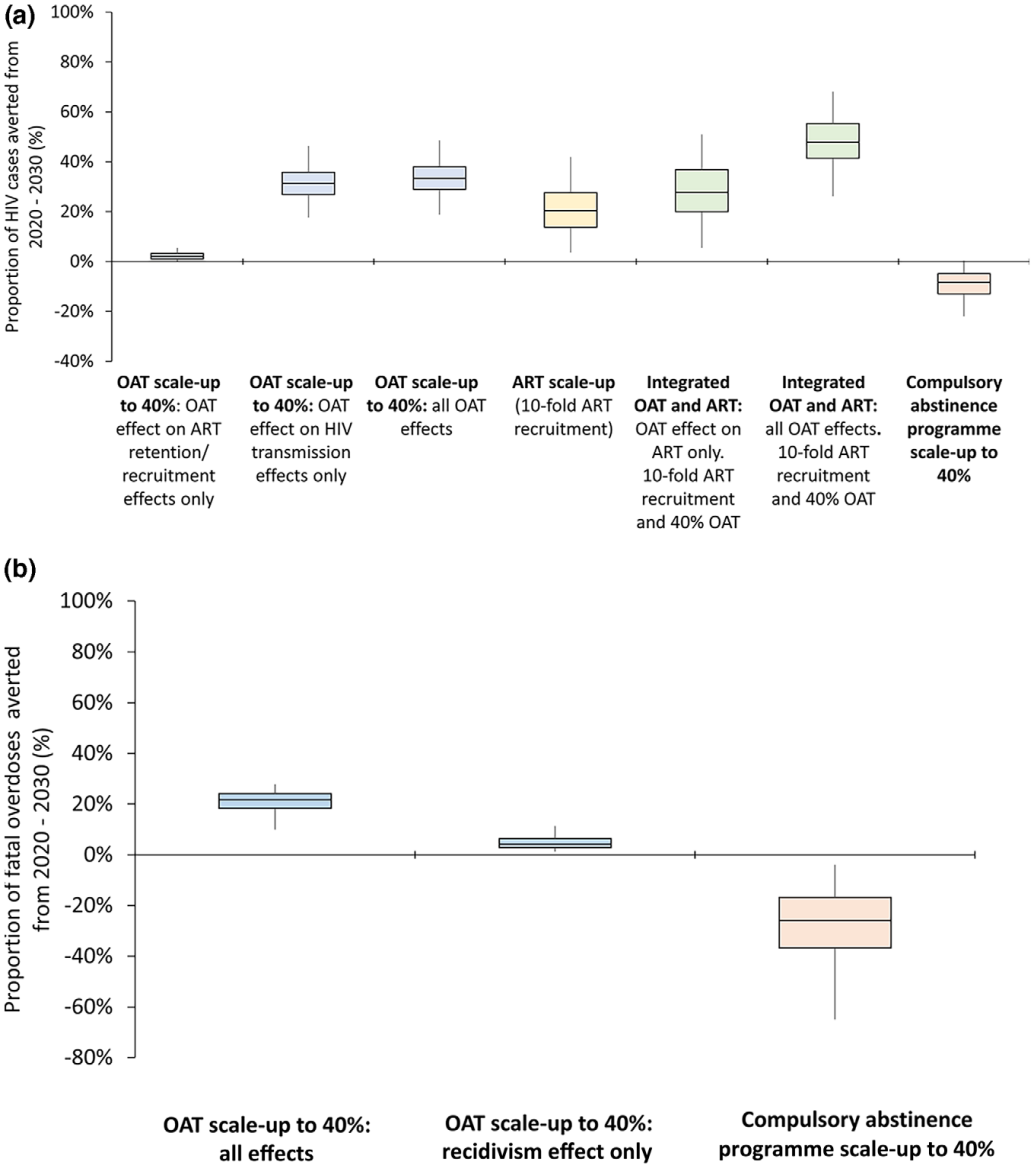
Proportion of HIV cases **(A)** and fatal overdoses **(B)** from 2020 to 2030 among PWID in Tijuana compared to the base case scenario. Blue boxplots represent opioid agonist therapy (OAT) scale‐up to 40% coverage only; yellow boxplot represents 10‐fold increase in ART recruitment only; green boxplots represent opioid agonist therapy scale‐up to 40% coverage plus increase in ART recruitment by 10‐fold. Red boxplots represent scale‐up of compulsory abstinence programmes to 40% coverage (instead of opioid agonist therapy).

**Figure 3:**
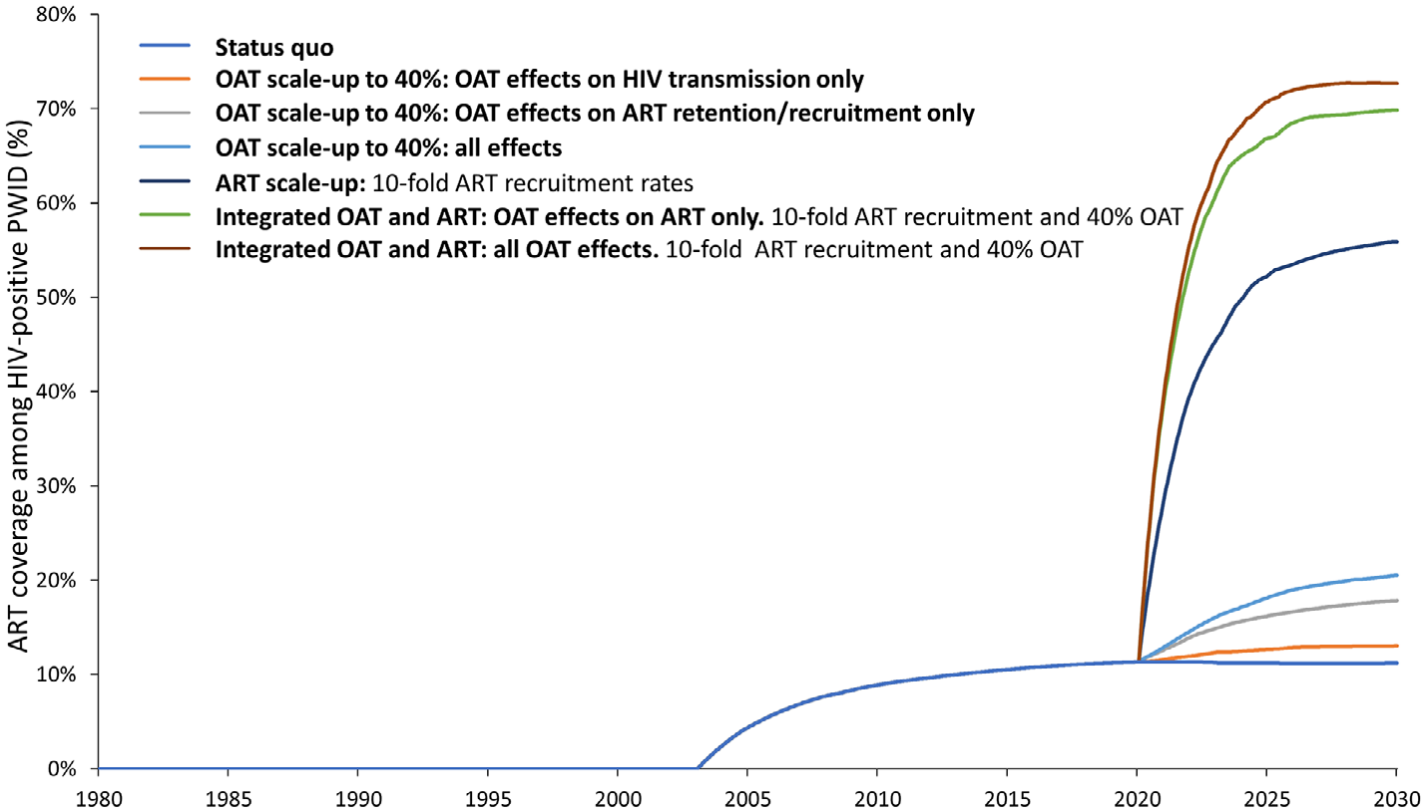
Trajectories of ART coverage levels under various opioid agonist therapy (OAT) and ART scale‐up scenarios.

In the status quo scenario, our model projected HIV prevalence to increase from 3.6% in 2020 to 4.8% in 2030. Figure 2 and Figure S6 show the impact of various intervention scenarios and components on new HIV infections (Figure 2A, Figure S6A) and fatal overdoses (Figure 2B, Figure S6B) among PWID over the next decade. When incorporating the full HIV prevention benefits of opioid agonist therapy (reducing HIV acquisition, increase in ART recruitment/retention, reduced reincarceration), opioid agonist therapy scale‐up among PWID could avert 31% (95% UI: 18%, 46%) of new HIV infections over the next decade (Figure 2A), and increase ART coverage from 11% to 21% among HIV‐positive PWID (Figure 3). Due to low baseline ART coverage, compared to the status quo, the independent effect of opioid agonist therapy on ART recruitment/retention only prevented 2% of new HIV infections over the next decade, with most of the opioid agonist therapy effect due to reduced HIV acquisition.

**Figure 4:**
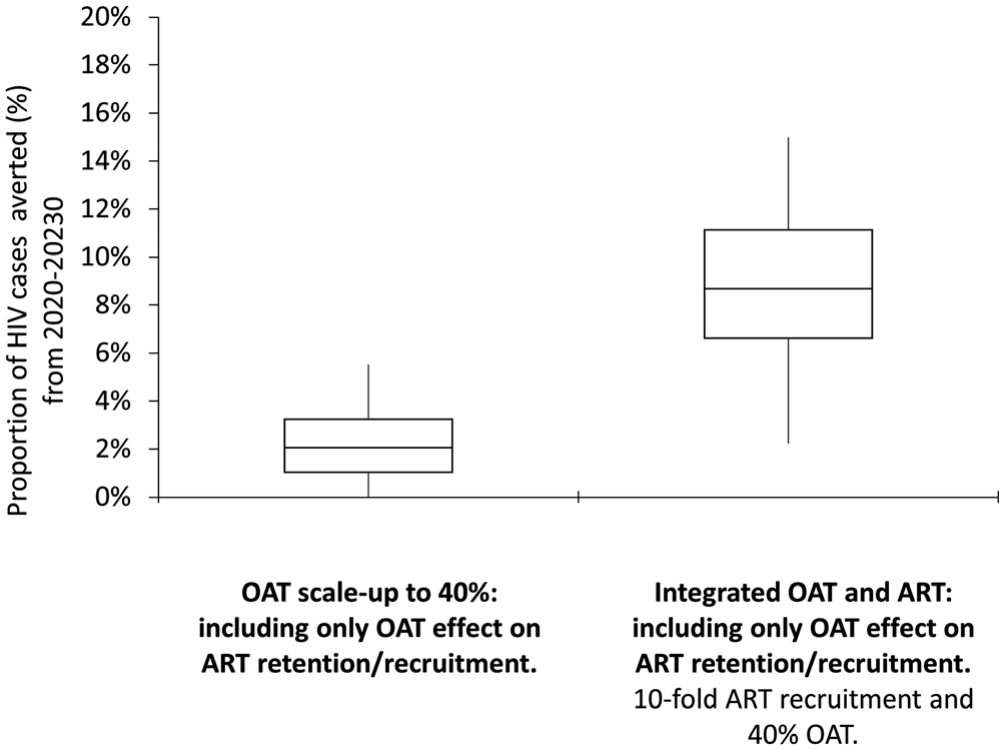
Proportion of HIV cases averted of opioid agonist therapy (OAT) effect on recruitment and retention to ART with and without ART scale‐up. In the scenario with opioid agonist therapy scale‐up only, the proportion of HIV cases averted was compared to the base case. In the scenario with opioid agonist therapy and ART scale‐up, the proportion of cases averted was compared to ART scale‐up only.

Increasing ART recruitment rates by 10‐fold elevated ART coverage from 11% in 2020 to 56% in 2030 among HIV‐positive PWID, and averted 20% (95% UI: 4%, 42%) of new HIV infections compared to the status quo over the next decade. With integrated opioid agonist therapy and ART scale‐up, ART coverage could reach 73% in 2030 and prevent 48% (95% UI: 26%, 68%) of new HIV infections in ten years if all opioid agonist therapy benefits were included compared to the status quo scenario. Importantly, synergistic effects were observed. When incorporating only the opioid agonist therapy effects on ART recruitment and retention, scaling‐up integrated opioid agonist therapy and ART (achieving 70% ART coverage among HIV‐positive PWID) averted 9% more (95% UI: 2% to 15%) HIV cases (Figure 4) compared to ART scale‐up alone (achieving 56% coverage). Conversely, scaling‐up compulsory abstinence programmes to 40% coverage (as opposed to opioid agonist therapy) could cause more HIV infections (−8% (95% UI: −22%, 0%)), due to increased risk of syringe sharing among individuals who had experience with compulsory abstinence programmes (Figure 2A) compared to the status quo.

The differential impact of opioid agonist therapy versus compulsory abstinence programme on fatal overdoses is shown in Figure 2B. With 40% opioid agonist therapy coverage among PWID, 22% (95% UI: 10%, 28%) of fatal overdoses could be averted over the next decade. Nearly 20% of this effect could be due to the indirect effect of opioid agonist therapy on reducing reincarceration. Conversely, compared to the status quo, scale‐up of compulsory abstinence programmes could result in more overdoses (−26%, 95% UI: −65%, −4%).

### 3.1 Sensitivity analyses

Overall impact of integrating OAT with ART on HIV and overdose outcomes was robust. Projections of HIV and overdoses averted deviated <15% with variations in OAT duration and effect estimates compared to the status quo scenario assumptions (Figure S7). Greater impact was achieved through longer duration OAT, or ‘best case’ scenario effect estimates. The projected impact of compulsory abstinence programme scale‐up was less sensitive to uncertainty in HIV effect estimates (<10% relative difference using best or worst case effect estimates), but moderately sensitive to uncertainty in overdose effect (16% fewer overdoses caused using “best‐case” effect estimates, 27% more overdoses caused using “worst‐case” effect estimates, compared to the baseline scenario.

## 4 Discussion

As HIV and overdose are two critical health risks facing PWID, we modelled the potential synergies of an integrated HIV and harm reduction response on HIV incidence and overdose among PWID in Tijuana, Mexico. Over the next decade, integrated scale‐up of ART (ten‐fold recruitment) and opioid agonist therapy (to 40% among PWID) could avert half of new HIV cases and one‐fifth of fatal overdoses among PWID. With concomitant scale‐up of ART recruitment, integration of opioid agonist therapy could interact synergistically by improving ART recruitment and retention, resulting in enhanced ART coverage and averting more HIV infections than if opioid agonist therapy were scaled up alone. Conversely, adopting non‐evidence based approaches to treat opioid use disorder, such as police operations that force PWID to compulsory abstinence programmes, could negatively impact HIV and overdose outcomes [39]. Within the context of drug policy reform, this finding underscores the importance for policymakers to ensure that HIV prevention and care is integrated with evidence‐based drug treatment programmes such as opioid agonist therapy to maximize impact and realize synergies (see Box 1).

Box 1 **Policy Implications of integrating HIV and harm reduction response on HIV and overdose among PWID in Tijuana**

- **Ensure provision of high‐quality, accessible, affordable opioid agonist treatment.** Increasing opioid agonist therapy coverage must be accompanied by other measures that include improving accessibility of opioid agonist therapy through increasing the number and geographical coverage of opioid agonist therapy providers; lowering opioid agonist therapy costs; disseminating information about the benefits of opioid agonist therapy to policymakers, health professionals and law enforcement; establishing a referral system within healthcare settings for people with opioid use disorder and guaranteeing protection of human rights. Greater knowledge of the benefits of these programmes at the community level could reduce stigma and reduce police harassment outside opioid agonist therapy clinics.
- **Ensure high coverage of HIV antiretroviral therapy.** Only a small proportion of PWID are receiving ART in Tijuana, in part because individuals are unaware of their infection and the ART treatment centre is not centrally located. Improvements along the HIV treatment cascade for PWID could be substantially enhanced if opioid agonist therapy and ART services were integrated and provided in a location near to where PWID live or gather.
- **Concomitant scale‐up of opioid agonist therapy and ART provision can provide synergistic benefits on HIV and overdose.** Our modelling highlights how opioid agonist therapy and ART can act synergistically by opioid agonist therapy further improving ART recruitment and retention, resulting in enhanced ART coverage compared to if only ART is scaled‐up in isolation. Opioid agonist therapy also prevents reincarceration, which can further prevent HIV and overdose. These benefits are in addition to direct benefits of opioid agonist therapy on prevention of overdose, HIV and hepatitis C virus (HCV).
- **Direct funding or referral to evidence‐based drug treatment programmes over non‐evidenced‐based compulsory abstinence programmes.** The government should reconsider any funding and referral to non‐evidenced based compulsory abstinence programmes as modelling indicates these programmes might cause more HIV infections and drug‐related deaths due to the increased risk of syringe sharing and overdose among individuals who are released from these sites.
- **Scale‐up complementary harm reduction services.** While not directly included in our modelling analyses, other harm reduction services will complement opioid agonist therapy and ART provision. The withdrawal of funding from the Global Fund has severely restricted needle and syringe programme provision, and scale‐up in access, provision and geographical coverage through formal services and in the community through NGO services and outreach programmes is required to prevent HIV and HCV. Naloxone is not currently available; free distribution and training on administration to health providers, police, first contact persons and PWID are critical to preventing opioid overdose deaths. Establishment of safe injection rooms could also have dual benefits on reducing overdose and preventing HIV.
- **Epidemic modelling can aid policymakers in understanding synergies and multiple benefits of integrated HIV care to inform decision making and resource allocation.** Models can synthesize data on intervention effects and synergies for multiple health outcomes to ensure the full public health impact of integrated care for PWID are understood, to inform evidence‐based decision making and resource allocation.

Despite the potential benefits of an integrated strategy, numerous challenges exist to implementation in Tijuana. First, opioid agonist therapy is prohibitively expensive for PWID. While one 80 mg methadone dose can cost between 3 and 6 USD [40], daily adherence would be difficult as most PWID in Tijuana earn less than $7 per day [6]. Second, police have harassed and arrested PWID outside of treatment centres [41], discouraging their willingness to seek care. For this reason, we partnered with the Tijuana police department to train officers on the benefits of harm reduction and align policing with public health [42]. However, with few available treatment slots and economic barriers to entry [43], it is unlikely police‐initiated referral to evidence‐based drug treatment will translate to successful enrolment and adherence. Third, although ART is free under the Mexican health care system, barriers to uptake and adherence remain. The main HIV clinic is approximately 25 km from the epicentre of HIV in Tijuana, and PWID experience stigma and discrimination which limit access [44]. To address these barriers, a binational student‐run free clinic was established in 2012 in a dense HIV and PWID area to enhance access to HIV testing, treatment and prevention services for key populations [45]. Additionally, local NGOs have provided care to PWID that did not satisfy the requirements to be treated at public services, but logistical difficulties arose around the collection and processing of laboratory samples for monitoring. Full integration of ART and opioid agonist therapy services will require changes to how ART is currently accessed. Greater coordination between opioid agonist therapy and HIV treatment providers will be needed to reach the 90‐90‐90 targets, which the Mexican government has committed to achieving [35]. By increasing ART recruitment 10‐fold and reaching 40% opioid agonist therapy coverage, ART coverage among PWID could reach these targets. However, this is likely optimistic given recent funding cuts to civil society organizations and centralization of ART procurement which has resulted in shortages [4].

Finally, while compulsory abstinence programmes in Tijuana are heterogeneous in terms of quality of care and abuse they inflict [46, 47], some of these centres imperfectly fill numerous unmet needs among PWID and their families, including housing and support in times of crisis [47, 48]. Integration of services among this marginalized population will need to address these key structural issues. Models in which opioid agonist therapy is provided in outpatient settings alongside centres offering shelter and support (including through religious, mutual and 12 steps models) should be considered. Data on patient outcomes, especially among those living with HIV and on ART, should also be rigorously collected and monitored. Furthermore, economic evaluations are warranted to determine the value‐for‐money (or lack thereof) of these programmes.

A strength of our study was the incorporation of numerous interacting benefits of opioid agonist therapy on HIV, overdose, ART and incarceration. Our modelling was consistent with previous studies indicating that scale‐up of harm reduction such as opioid agonist therapy can have substantial benefits on preventing HIV [16, 49, 50], and fatal overdoses among PWID [49]. It was also consistent with findings that opioid agonist therapy can improve the HIV prevention benefits of ART [51]. While we did not model the impact of opioid agonist therapy in incarcerated settings, previous modelling has shown substantial benefits of scaling up these services in prisons [52].

As with all modelling, there is uncertainty in several parameter estimates. First, we note that many opioid agonist therapy effect estimates were based on findings from global systematic reviews/meta‐analyses of studies primarily from high‐income settings which might not be generalizable to settings like Tijuana. We have documented structural barriers to opioid agonist therapy in Tijuana, yet once accessed, services are high quality and provide comprehensive support, such as legal services and psychosocial counselling [40]. Second, data from Tijuana indicate an association between recent compulsory abstinence programme attendance and non‐fatal overdose, which we assumed was similar for fatal overdose due to lack of data. Additionally, historic exposure to compulsory abstinence programmes is associated with receptive syringe sharing. Both these associations require further studies to confirm causal pathways. Third, we used setting‐specific estimates for fatal overdoses, but this rate is highly uncertain due to lacking cause of death information and underreporting of overdoses [8]. However, our results in terms of proportions of overdoses averted should be robust to uncertainty in the absolute overdose rate. Fourth, we neglected polysubstance use, but 15% of PWID in Tijuana report injecting both opioids and stimulants, which could alter the effect of opioid agonist therapy [53]. We purposely limited opioid agonist therapy scale‐up to 40% coverage among PWID to account for a proportion who may be ineligible. Fifth, our model was calibrated to HIV data indicating increasing HIV prevalence from 2005 to 2014. Thus, findings are conditional on a growing epidemic. Furthermore, our model did not incorporate other harm reduction interventions such as needle/syringe programmes or naloxone distribution, as our focus was primarily on exploring synergies between harm reduction and HIV services, as observed through opioid agonist therapy on ART. However, provision of comprehensive harm reduction services and scale‐up of needle/syringe programmes is urgently required in Tijuana to prevent HIV and HCV [54, 55]. Lastly, our results did not incorporate potential benefits of prevention interventions on transmission to non‐injecting sexual partners, which would increase impact. Box 2 outlines the research agenda for integrating opioid agonist therapy with HIV treatment services.

Box 2 **Research Agenda of Integrating Opioid Agonist Therapy with HIV Treatment Services**

- Modelling and economic analyses can elucidate the most efficient way to allocate resources across harm reduction and HIV treatment and prevention services to maximize health gains. Further work is needed to include additional synergistic benefits on other outcomes such as hepatitis C virus (HCV).
- Similarly, modelling can estimate the synergistic impact of structural interventions, such as drug law reform and police education programmes to align policing with public health. These programmes could further improve referral to opioid agonist therapy and ART, reduce incarceration of PWID, and reduce injection risk among PWID, further enhancing HIV and overdose prevention efforts.
- Our data indicate exposure to compulsory abstinence programme is associated with receptive syringe sharing and overdose. Further research is warranted to understand whether there is also an interaction between compulsory abstinence programmes and other HIV risks or ART provision.
- Despite the potential benefits of an integrated strategy to address HIV and broader health harms among PWID, numerous challenges exist to its implementation. Research on interventions addressing economic and stigma‐related barriers to HIV prevention, care and treatment are warranted.
- Full integration of ART and opioid agonist therapy services will require changes to how ART is currently accessed and greater coordination between opioid agonist therapy and HIV providers. Service delivery questions will need to be addressed through health system research.

## 5 Conclusions

Our model indicates that integrating HIV with evidence‐based drug treatment services provides opportunities for synergies across multiple health outcomes affecting PWID in Tijuana. Policymakers need to understand the impact of scaling‐up evidence‐based programmes versus non‐evidence based, especially within the context of reduced funding and drug policy reforms aligned with harm reduction. To realize the full potential of these services, structural and economic barriers will need to be removed and calls from the United Nations should be heeded to cease detainment in compulsory abstinence programmes [56].

## Competing interests

NM has received unrestricted research grants from Gilead and Merck unrelated to this work. All other authors declare no competing interests.

## Authors’ Contributions

JC and NM conceptualized the study analyses and wrote the first draft of the manuscript. AB and JC developed the model. JC generated model results and figures. CM and AV conducted literature reviews to help with model parameterization. CR assisted with modelling compulsory abstinence programmes. GR, MEM and SS analysed the results and aided in manuscript drafting. All authors critically reviewed and approved the manuscript for submission.

## Supporting information


**Figure S1**. Schematic of extensions to model by Borquez et al. Red shaded compartments display HIV disease progression and recruitment onto ART; orange shaded compartments display recruitment onto/off opioid agonist therapy; blue shaded compartments display movement between incarcerated states.

**Figure S2.** Proportion of PWID on antiretroviral therapy (ART). ART was assumed to have been available in 2003 and was calibrated to vary uniformly between 2% and 18% in 2012. 95% uncertainty bounds are represented by the grey shaded region.

**Figure S3.** Median and 95% uncertainty interval of base case model projections of HIV prevalence **(A)** overall; **(B)** among male PWID only; **(C)** and among female PWID only. Solid lines represent median and dashed lines represent 95% uncertainty interval bounds. Red triangle denotes calibration point and error bars represent 95% confidence intervals.

**Figure S4.** Median and 95% uncertainty interval of base case model projections of HIV incidence **(A)** among male PWID only; **(B)** and among female PWID only. Solid lines represent median and dashed lines represent 95% uncertainty interval bounds. Red triangle denotes calibration point and error bars represent 95% confidence intervals.

**Figure S5.** Proportion of PWID on opioid agonist therapy **(A)** or compulsory abstinence programmes **(B)** after scale‐up starting in 2020.

**Figure S6.** Scatterplot of impact of each simulation compared to the status quo scenario for **(A)** HIV cases averted and **(B)** fatal overdoses averted from 2020 to 2030.

**Figure S7.** Results from sensitivity analyses showing relative change in median proportion of new HIV infections and overdoses averted compared to the scenario with full benefits of OAT on HIV and overdose outcomes **(A)** and compulsory abstinence programme (panel B). In **(A)**, duration of OAT was varied from six months (short duration) to two years (long duration) and the effect of OAT was increased or decreased to the upper and lower bounds of the 95% confidence limits of parameter values for its effect on reducing both HIV transmission and fatal overdose. Similarly, in **(B)**, the effect of the compulsory abstinence programme was increased or decreased to the upper and lower bounds of the 95% confidence limits for its effect on increasing risk of HIV transmission and fatal overdose.

**Table S1.** Parameters informing the HIV transmission model among PWID in Tijuana, for more details see Borquez et al

**Table S2.** Model calibration data among people who inject drugs in Tijuana, Mexico

**Data S1.** Supplementary information.
