# Supplementary material for: Modelling integrated antiretroviral treatment and harm reduction services on HIV and overdose among people who inject drugs in Tijuana, Mexico
Source: J Int AIDS Soc. 2020 Jun 19;23(Suppl 1):e25493. doi: 10.1002/jia2.25493 (PMC7305416; doi:10.1002/jia2.25493)
Supplement: Supplementary file 1 — Figure S1. Schematic of extensions to model by Borquez et al. Red shaded compartments display HIV disease progression and recruitment onto ART; orange shaded compartments display recruitment onto/off opioid agonist therapy; blue shaded compartments display movement between incarcerated states. [file JIA2-23-e25493-s001.docx]

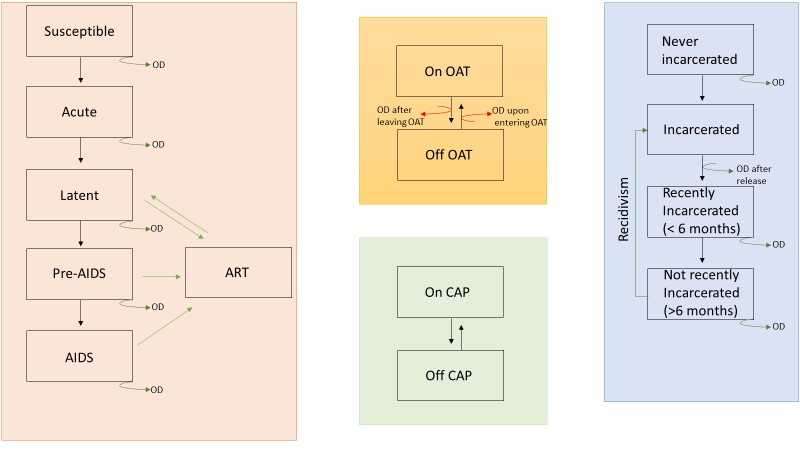


**Figure S1.** Schematic of extensions to model by Borquez et al. Red shaded compartments display HIV disease progression and recruitment onto ART; orange shaded compartments display recruitment onto/off opioid agonist therapy; blue shaded compartments display movement between incarcerated states.
