# Supplementary material for: Modelling integrated antiretroviral treatment and harm reduction services on HIV and overdose among people who inject drugs in Tijuana, Mexico
Source: J Int AIDS Soc. 2020 Jun 19;23(Suppl 1):e25493. doi: 10.1002/jia2.25493 (PMC7305416; doi:10.1002/jia2.25493)
Supplement: Supplementary file 2 — Figure S2. Proportion of PWID on antiretroviral therapy (ART). ART was assumed to have been available in 2003 and was calibrated to vary uniformly between 2% and 18% in 2012. 95% uncertainty bounds are represented by the grey shaded region. [file JIA2-23-e25493-s002.docx]

**Figure S2.** Proportion of HIV-infected PWID on antiretroviral therapy (ART). ART was assumed to have been available in 2003 and was calibrated achieve 2% - 18% coverage in 2012. 95% uncertainty bounds are represented by the gray shaded region.
