# Supplementary material for: Modelling integrated antiretroviral treatment and harm reduction services on HIV and overdose among people who inject drugs in Tijuana, Mexico
Source: J Int AIDS Soc. 2020 Jun 19;23(Suppl 1):e25493. doi: 10.1002/jia2.25493 (PMC7305416; doi:10.1002/jia2.25493)
Supplement: Supplementary file 4 — Figure S4. Median and 95% uncertainty interval of base case model projections of HIV incidence (A) among male PWID only; (B) and among female PWID only. Solid lines represent median and dashed lines represent 95% uncertainty interval bounds. Red triangle denotes calibration point and error bars represent 95% confidence intervals. [file JIA2-23-e25493-s004.docx]

**
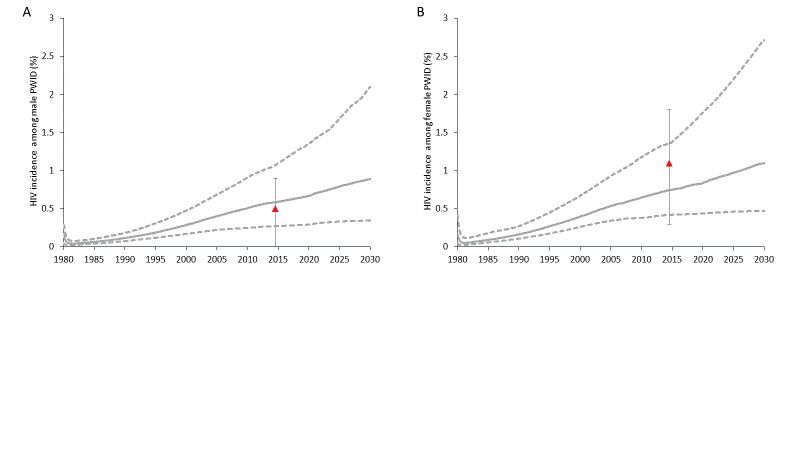
**

**Figure S4.** Median and 95% uncertainty interval of base case model projections of HIV incidence (A) among male PWID only; (B) and among female PWID only. Solid lines represent median and dashed lines represent 95% uncertainty interval bounds. Red triangle denotes calibration point and error bars represent 95% confidence intervals
