# Supplementary material for: Modelling integrated antiretroviral treatment and harm reduction services on HIV and overdose among people who inject drugs in Tijuana, Mexico
Source: J Int AIDS Soc. 2020 Jun 19;23(Suppl 1):e25493. doi: 10.1002/jia2.25493 (PMC7305416; doi:10.1002/jia2.25493)
Supplement: Supplementary file 5 — Figure S5. Proportion of PWID on opioid agonist therapy (A) or compulsory abstinence programmes (B) after scale‐up starting in 2020. [file JIA2-23-e25493-s005.docx]

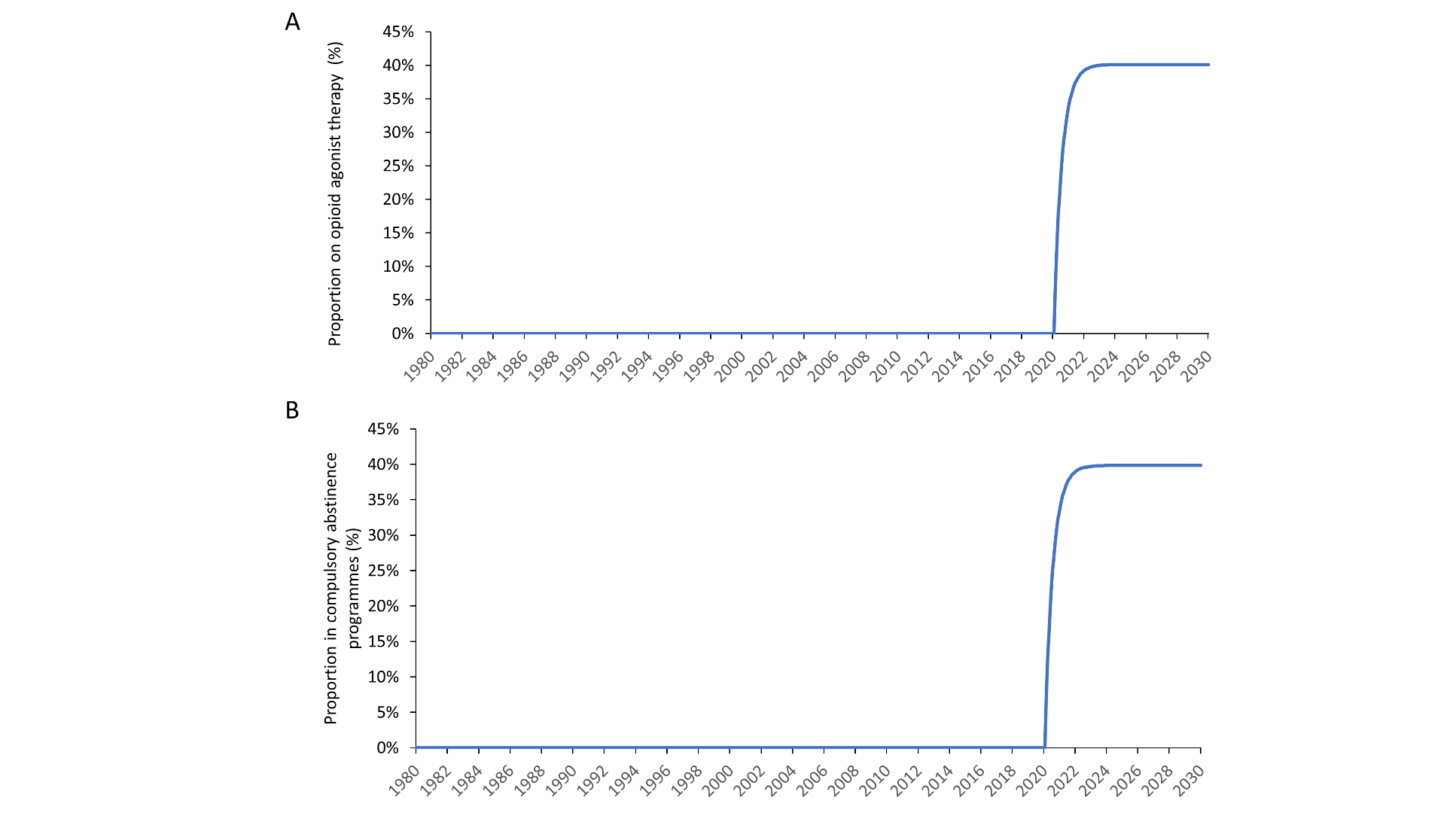


**Figure S5.** Proportion of PWID on opioid agonist therapy (A) or compulsory abstinence programmes (B) after scale-up starting in 2020.
