# Supplementary material for: Modelling integrated antiretroviral treatment and harm reduction services on HIV and overdose among people who inject drugs in Tijuana, Mexico
Source: J Int AIDS Soc. 2020 Jun 19;23(Suppl 1):e25493. doi: 10.1002/jia2.25493 (PMC7305416; doi:10.1002/jia2.25493)
Supplement: Supplementary file 6 — Figure S6. Scatterplot of impact of each simulation compared to the status quo scenario for (A) HIV cases averted and (B) fatal overdoses averted from 2020 to 2030. [file JIA2-23-e25493-s006.docx]

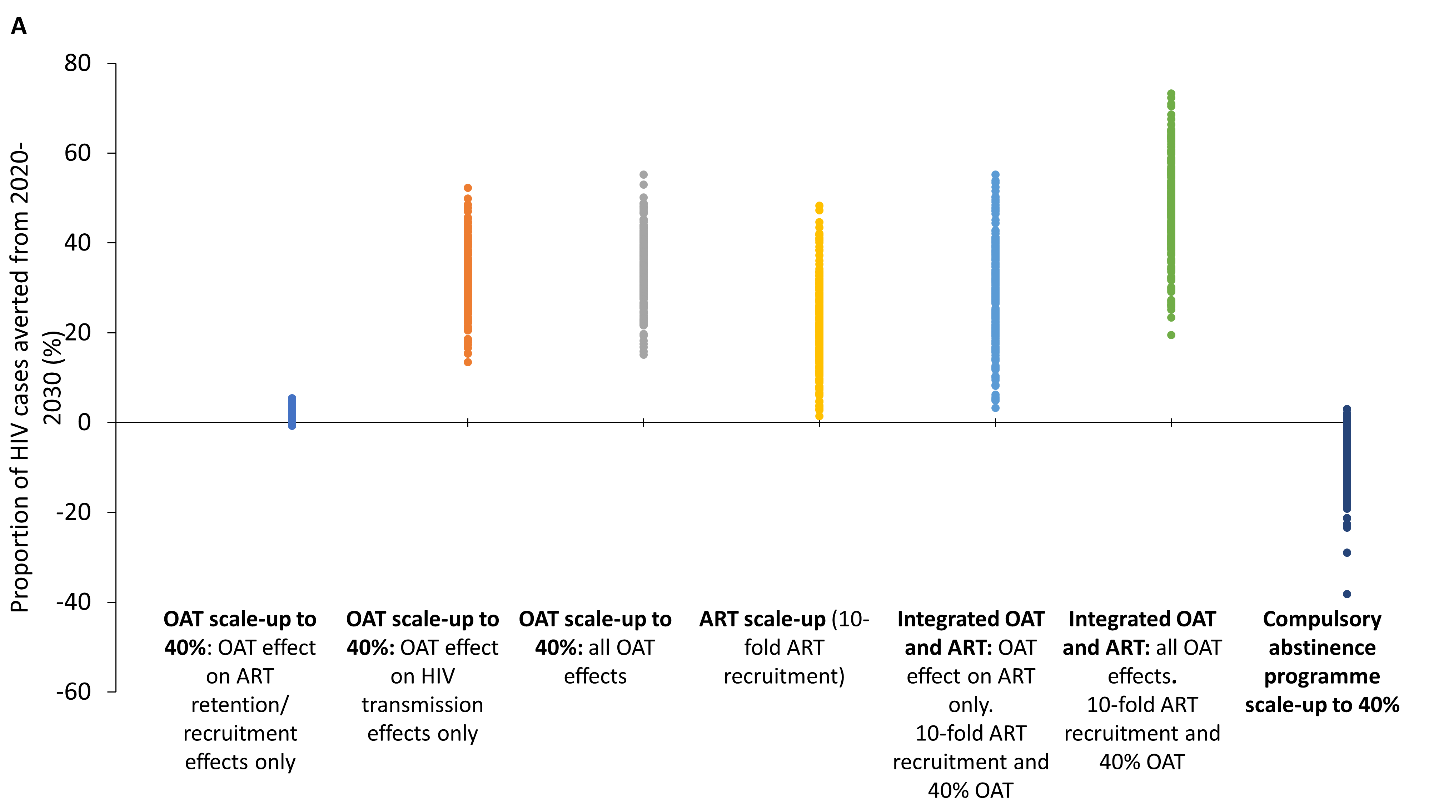


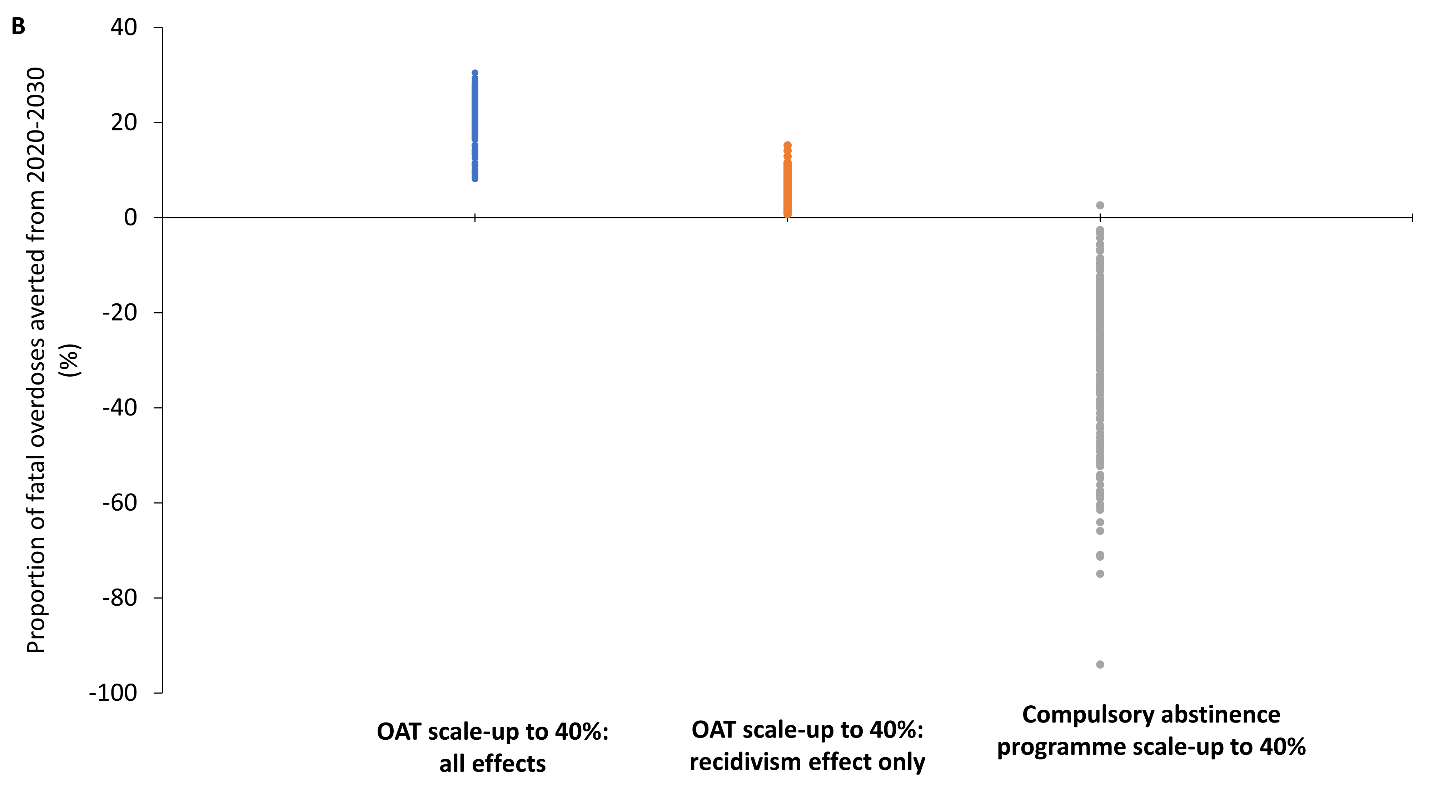


**Figure S6.** Scatterplot of impact of each simulation compared to the status quo scenario for (A) HIV cases averted and (B) fatal overdoses averted from 2020-2030.
