# Supplementary material for: Modelling integrated antiretroviral treatment and harm reduction services on HIV and overdose among people who inject drugs in Tijuana, Mexico
Source: J Int AIDS Soc. 2020 Jun 19;23(Suppl 1):e25493. doi: 10.1002/jia2.25493 (PMC7305416; doi:10.1002/jia2.25493)
Supplement: Supplementary file 7 — Figure S7. Results from sensitivity analyses showing relative change in median proportion of new HIV infections and overdoses averted compared to the scenario with full benefits of OAT on HIV and overdose outcomes (A) and compulsory abstinence programme (panel B). In (A), duration of OAT was varied from six months (short duration) to two years (long duration) and the effect of OAT was increased or decreased to the upper and lower bounds of the 95% confidence limits of parameter values for its effect on reducing both HIV transmission and fatal overdose. Similarly, in (B), the effect of the compulsory abstinence programme was increased or decreased to the upper and lower bounds of the 95% confidence limits for its effect on increasing risk of HIV transmission and fatal overdose. [file JIA2-23-e25493-s007.docx]

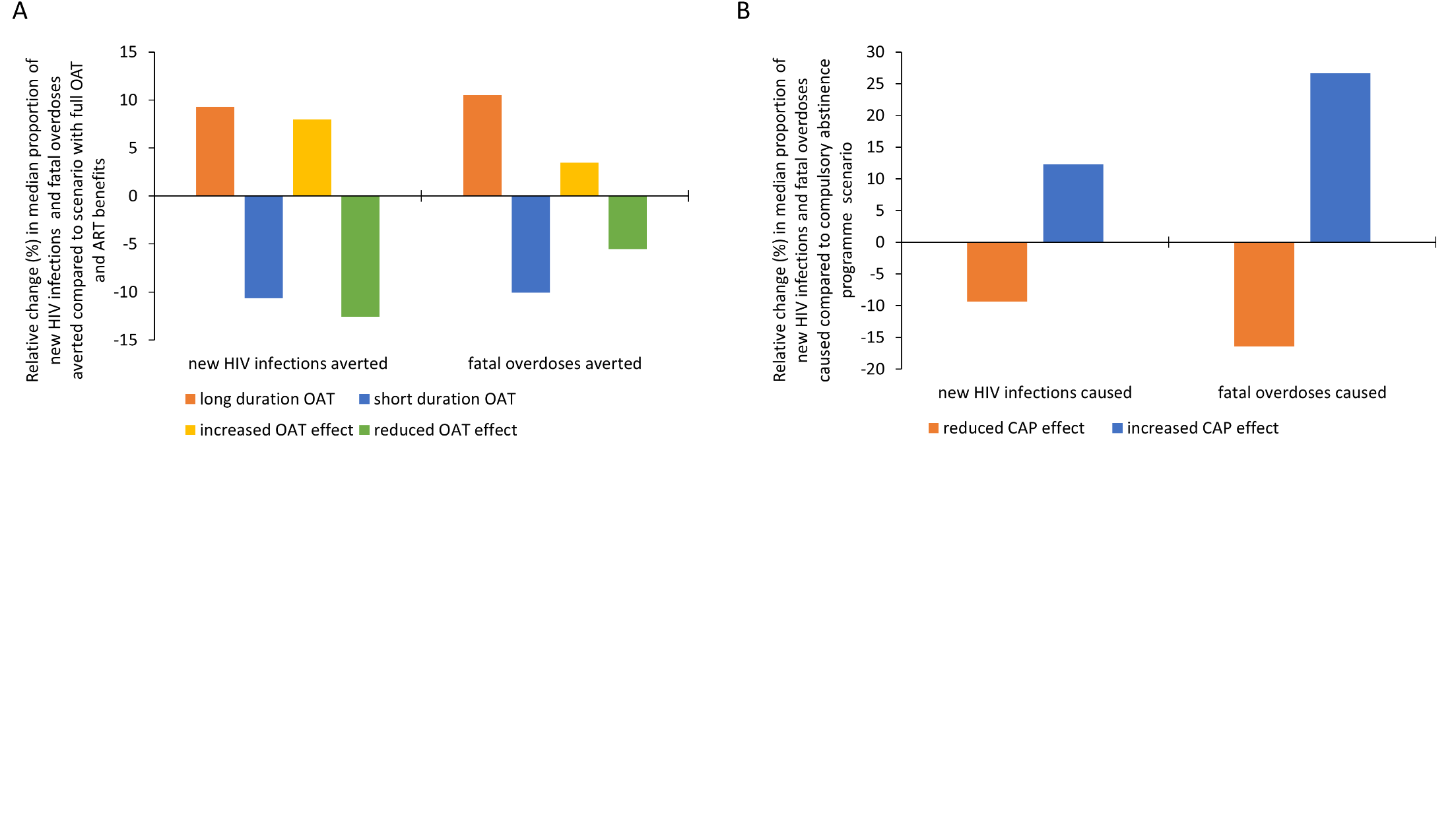

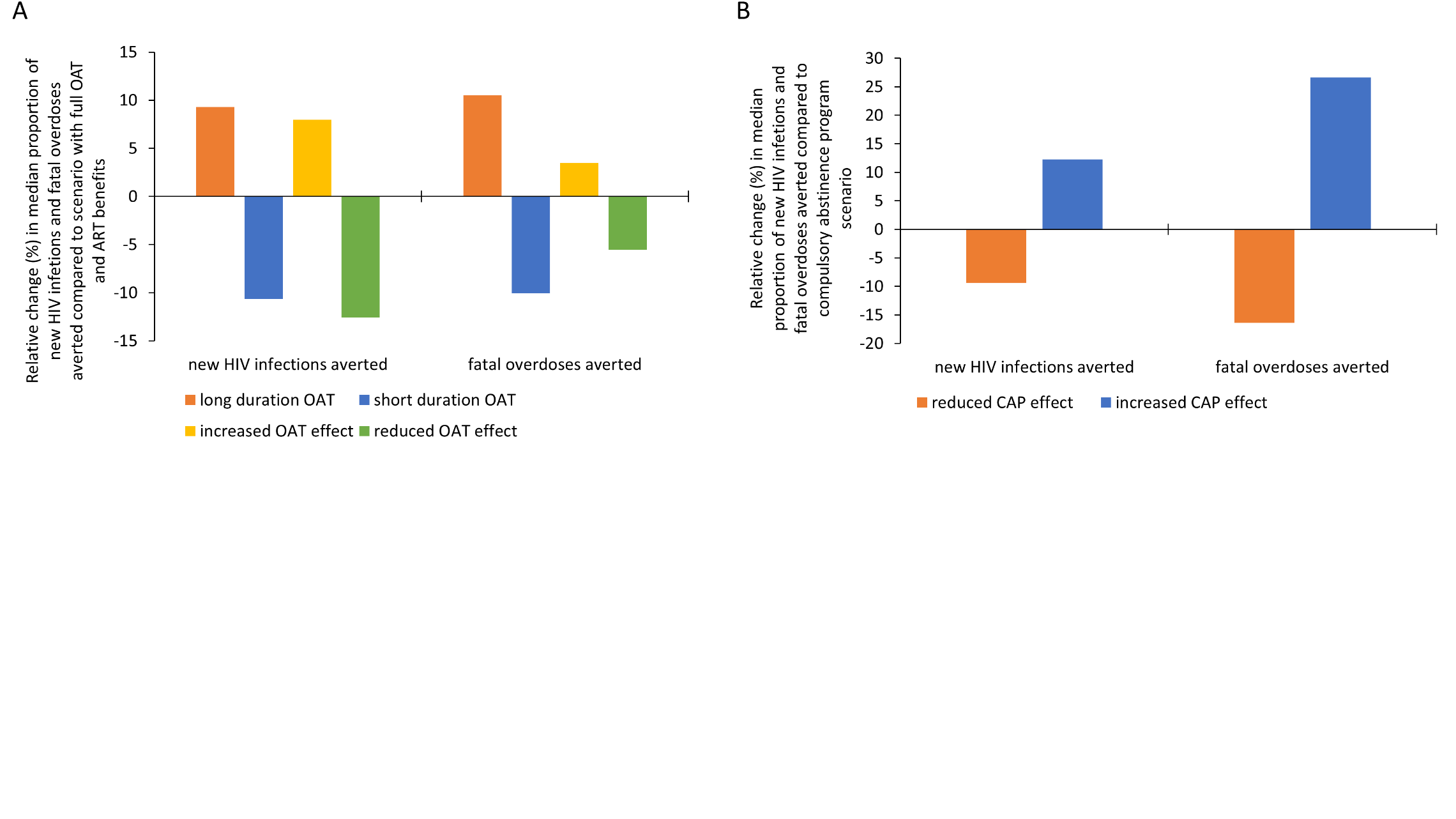


**Figure S7: Results from sensitivity analyses showing relative change in median proportion of new HIV infections and overdoses averted or caused compared to the scenario with full benefits of OAT on HIV and overdose outcomes (panels A and B) and compulsory abstinence programme (panel C).** In panel A, duration of OAT was varied from 6 months (short duration) to 2 years (long duration). In panel B, the effect of OAT was increased or decreased to the upper and lower bounds of the 95% confidence limits of parameter values for its effect on reducing both HIV transmission and fatal overdose. Similarly, in panel C, the effect of the compulsory abstinence programme was increased or decreased to the upper and lower bounds of the 95% confidence limits for its effect on increasing risk of HIV transmission and fatal overdose.
