# Supplementary material for: Modelling integrated antiretroviral treatment and harm reduction services on HIV and overdose among people who inject drugs in Tijuana, Mexico
Source: J Int AIDS Soc. 2020 Jun 19;23(Suppl 1):e25493. doi: 10.1002/jia2.25493 (PMC7305416; doi:10.1002/jia2.25493)
Supplement: Supplementary file 8 — Table S1. Parameters informing the HIV transmission model among PWID in Tijuana, for more details see Borquez et al [file JIA2-23-e25493-s008.docx]

**Table S1. Parameters informing the HIV transmission model among PWID in Tijuana, for more details see Borquez et al.**

| **Parameters (symbol)** | **Point Estimate (Sampled Range)**  Men Women | **Sampling Distribution** | **Posterior distribution**  **95% uncertainty interval**  Men Women | **Source** |
| --- | --- | --- | --- | --- |
| **Demographic** |  |  |  |  |
| Size of PWID population (N) | 10,000 | Beta |  | [5]; El Cuete III data |
| Proportion of PWID who are men at entry (κ) | 0·80 (0·7-0·9) |  | 0.81 (0.74 – 0.86) | [6]; El Cuete III data |
| Mean duration of injecting among PWID (years) (τ) | 12-25 5-20 | Uniform | 13-25 7-20 | [7]; El Cuete IV data |
| Mean mortality rate among PWID per year (Ι) | 0·03 |  |  | [6]; El Cuete III data |
|  |  |  |  | definition |
| Mean rate of exiting prison among PWID per year (1/φ) | 3·1 (0-7) 2·9 (0-7) | Truncated  normal | 3.6 (0.6 – 8) 3.0 (0.3 – 8) | [6]; El Cuete III data |
| Rate of exiting recent incarceration per year (θi) | 2 2 | Fixed |  | Model |
|  |  |  |  | definition |
| Proportion of PWID incarcerated before starting injecting (γ) | 82% (79%-92%) 33% (19%-61%) | fitted | 84%(79%-92%) 28%(19%-59%) | Fitted to El Cuete IV data using cohort submodel, in [1] |
| Primary incarceration rate per year (ς) | 0·018(0-0·047) 0·034 (0·001-0·055) | fitted | 0.19(0.0 – 0.049) 0.04(0.0-0.059) | Fitted to El Cuete IV data using cohort submodel in [1] |
| Reincarceration rate per year | 0·27 (0·08-0·46) 0·2 (0·03-0·40) | Truncated  normal | 0.27 (0.08-0.44) 0.2(0.04-0.43) | [7]; El Cuete IV data |
|  |  |  |  |  |
| **Behavioural** |  |  |  |  |
| Relative risk of receptive syringe sharing among recently released from prison vs not (RR^inc^) | 1.30 (1.15-1.46) | Lognormal | 1.30 (1.19 – 1.50) | [7]; El Cuete IV data |
| Relative risk of receptive syringe sharing among recently exposed vs. not recently exposed to syringe confiscation by the police (RR^SC^) | 1.16 (1.03-1.29) | Lognormal | 1.16 (1.01-1.32) | [7]; El Cuete IV data |
| Rate of OAT cessation per year (ϖ) | 1 |  |  | [8, 9] |
| Number of sharing events in the past year among PWID (C) | 10-472 | Uniform | 11-443 | [10]; El Cuete III data |
| Number of stable sexual partners per year (sstable) | 0·4-0·5 0·65-0·75 | Uniform | 0.4-0.5 0.65-0.75 | [7]; El Cuete IV data |
| Number of casual sexual partners per year (scasual) | 0-4 0-8 | Uniform | 0-4 0-8 | [7]; El Cuete IV data |
| Number of commercial partners per year (scom) | 0-0·5 0-20 | Uniform | 0.0.5 1-20 | [7]; El Cuete IV data |
| Number of sex acts per stable partner per year (astable) | 25-100 | Uniform | 28-98 | [7]; El Cuete IV data |
| Number of sex acts per casual partner per year (acasual) | 1-25 | Uniform | 2-24 | [7]; El Cuete IV data |
| Number of sex acts per commercial partner per year (acom ) | 1-12 | Uniform | 1-12 | [7]; El Cuete IV data |
| Frequency of condom use with stable partner (kstable) | 0.04-0.12 | Uniform | 0.04-0.12 | [7]; El Cuete IV data |
| Frequency of condom use with casual partner(kcasual) | 0.19-0.31 | Uniform | 0.19-0.31 | [7]; El Cuete IV data |
| Frequency of condom use with commercial (kcom) )partner(proportion) | 0.24-0.38 | Uniform | 0.24-0.37 | [7]; El Cuete IV data |
| HIV prevalence among non-PWID sexual partners (P) | Linear increase to reach 0·5-1% in 2005, stable thereafter | Uniform |  | [4] |
| Proportion of sexual partnerships among PWID (ϖ) | 1.5% 10% |  |  | [1] |
| **Biological** |  |  |  |  |
| Number of HIV positive PWID at the start of the epidemic (seed) | 4 (2-6) 2(0·5-4) | Truncated normal | 4(1-6) 2(0-5) | Fitted |
| Average duration of acute HIV stage (months) (σ2) | 2·9 |  |  | [11] |
| Average duration of latent HIV stage (years) (σ3) | 8 |  |  | [11] |
| Average duration of pre-AIDS stage (months) (σ4) | 9 |  |  | [11] |
| Average duration of AIDS stage (months) (σ5) | 10 |  |  | [11] |
| Average duration of ART stage (years) (σ6) | 20 |  |  |  |
| HIV transmission probability through syringe sharing during the latent stage per receptive sharing contact (β3) | 0·0001-0·024 | Uniform | 0.0002-0.0092 | [12] |
| HIV transmission probability through sex during the latent stage per sex act (β sex 3) | 0·0006-0·0017 | Uniform | 0.0007-0.0017 |  |
| Relative increase in HIV transmission probability compared to latent stage during the: |  |  |  |  |
| Acute stage (RRh2) | 3-26 | Uniform | 3-25 | [11] |
| PreAIDS stage (RRh4) | 1-7 | Uniform | 1-7 | [11] |
| AIDS stage (RRh5) | 0 |  |  | [11, 13] |
