# Supplementary material for: Modelling integrated antiretroviral treatment and harm reduction services on HIV and overdose among people who inject drugs in Tijuana, Mexico
Source: J Int AIDS Soc. 2020 Jun 19;23(Suppl 1):e25493. doi: 10.1002/jia2.25493 (PMC7305416; doi:10.1002/jia2.25493)
Supplement: Supplementary file 9 — Table S2. Model calibration data among people who inject drugs in Tijuana, Mexico [file JIA2-23-e25493-s009.docx]

**Table S2. Model calibration data among people who inject drugs in Tijuana, Mexico**

| **Calibration values** | **Male PWID Point Value**  **95% CI** | **Female PWID Point Value**  **95% CI** | **Distribution** | **Source** |
| --- | --- | --- | --- | --- |
| HIV prevalence among PWID  2005 (overall) | 2.3% (1-5.3%) | | Beta | El Cuete II data |
| HIV prevalence among PWID  2006  2011 | 2.4% (1.3-3.6%)  3.5% (2.0-5.4%) | 5.4% (1.3-7.8%)  3.6% (2.0 – 6.5%) | Beta | [5]; El Cuete III and El Cuete IV data |
| HIV prevalence among ever incarcerated PWID in 2011 | 3.5% (1.7-5.4%) | 5.2% (1.7-8.8%) | Beta | [6]; El Cuete IV data |
| Relative HIV prevalence among ever versus never incarcerated PWID in 2011 | 1.1 (0.3 – 4.7) | 3.2 (0.7 – 15) | Normal | [6]; El Cuete IV data |
| HIV incidence among PWID in 2014 (per 100 person-years) | 0.5 (0.06 – 0.9) | 1.1 (0.3 – 1.8) | Poisson | [6]; El Cuete IV data |
| Proportion of new HIV infections among PWID attributable to sexual transmission | 0.45 (0.2-0.7) | | Normal | See HIV/syphilis coinfectio model in [1] |
| ART coverage among HIV-infected PWID in 2012 | 2-18% | | Uniform | Based on El Cuete data and data on a study linking key population to HIV treatment in Tijuana [7] |
