# Supplementary material for: Modelling integrated antiretroviral treatment and harm reduction services on HIV and overdose among people who inject drugs in Tijuana, Mexico
Source: J Int AIDS Soc. 2020 Jun 19;23(Suppl 1):e25493. doi: 10.1002/jia2.25493 (PMC7305416; doi:10.1002/jia2.25493)
Supplement: Supplementary file 10 — Data S1. Supplementary information. [file JIA2-23-e25493-s010.docx]

**Supplemental information**

**Model equations**

Full details on model structure can be found in the manuscript by Borquez et al. [1]. Here we describe only the extensions to the model that we used to generate the results. Briefly, the state variables that we modified are given by: $X_{i,r,h,o}\left( t \right).$ *t* is the time elapsed in the simulation; *i* is the sex (1=men, 2=women), *r* is the incarceration stage (1=never incarcerated, 2=currently incarcerated, 3=recently incarcerated (<6 months ago), 4=Not recently incarcerated (>6 months ago)), *h* is the HIV infection-status (1= susceptible, 2= acute infection, 3= latent infection, 4= pre-AIDS, 5=AIDS, 6=ART), and *o* is the intervention status (1=on intervention, 2 = not on intervention). An additional state related to syringe confiscation (s) was included in the original model however we did not modify this. We define intervention as being either admitted to OAT or CAP, with the corresponding relative measures of association dependent on the type of intervention (denoted as RR^HIVint^ or RR^ODint^ in the modelling equations below). For example, individuals on the OAT intervention would be less likely to acquire HIV (RR=0.46) but those on the CAP intervention would be more likely to engage in syringe sharing (RR=1.14). Incarcerated individuals were not eligible for OAT nor ART. Participants could be recruited onto the intervention (either OAT or CAP) and drop out of the intervention. We assumed duration of the intervention to be one year. *N* corresponds to the total population, where HIV and overdose related deaths were not replaced. The equations determining both the infection process and the movement through incarceration stages are presented below:

Never incarcerated

$$\frac{dX_{i,1,1,o}\left( t \right)}{dt}= \kappa_{i}\left( 1-\gamma_{i,r} \right)\omega_{i}\left[ lN+\sum_{i} \tau_{i}N_{i} \right]-{{RR}_{o}^{HIVint}\lambda}^{inj}X_{i,1,1,o}- \lambda^{sex}X_{i,1,1,o}{OAT}_{ODentry}\upsilon X_{i,1,2,1,o}+ {OAT}_{ODexit}\delta X_{i,1,1,o}-\left( ϛ_{i}+ \tau_{i}+ l+ \mu\right)X_{i,1,1,o}$$

$$\frac{dX_{i,1,2,o}\left( t \right)}{dt}= {{RR}_{o}^{HIVint}\lambda}^{inj}X_{i,1,1,o}+ \lambda^{sex}X_{i,1,1,o}+{(1-OAT}_{ODexit}) \delta X_{i,1,2,o}-(1-{OAT}_{ODentry})\upsilon X_{i,1,2,o}-\left( \sigma_{2}+ ϛ_{i}+ \tau_{i}+ l+ {RR}_{o}^{ODint}\mu\right)X_{i,1,2,o}$$

$$\frac{dX_{i,1,3,o}\left( t \right)}{dt}= {\sigma_{2}X}_{i,1,2,o}+{(1-OAT}_{ODexit}) \delta X_{i,1,3,o}+{RR}_{o}^{ARTdropout}{\psi_{1}X}_{i,1,6,o} {{- {RR}_{o}^{ARTrecruit} \rho}_{1}X}_{i,1,3,o}-{(1-OAT}_{ODentry})\upsilon X_{i,1,3,o}-\left( \sigma_{3}+ ϛ_{i}+ \tau_{i}+ l+ {RR}_{o}^{ODint}\mu\right)X_{i,1,3,o}$$

$$\frac{dX_{i,1,4,o}\left( t \right)}{dt}= {\sigma_{3}X}_{i,1,3,o}+(1-{OAT}_{ODexit}) \delta X_{i,1,4,o}-{{RR}_{o}^{ARTrecruit}\rho_{1}X}_{i,1,4,o}-{(1-OAT}_{ODentry})\upsilon X_{i,1,4,o}-\left( \sigma_{4}+ ϛ_{i}+ \tau_{i}+ l+ {RR}_{o}^{ODint}\mu\right)X_{i,1,4,o}$$

$$\frac{dX_{i,1,5,o}\left( t \right)}{dt}= {\sigma_{4}X}_{i,1,4,o}+{(1-OAT}_{ODexit}{) \delta}_{s}X_{i,1,1,5,o}-{{RR}_{o}^{ARTrecruit}\rho_{1}X}_{i,1,s,5,o}-{(1-OAT}_{ODentry})\upsilon_{i}X_{i,1,5,o}-\left( \sigma_{5}+ ϛ_{i}+ \tau_{i}+ l+ {RR}_{o}^{ODint}\mu\right)X_{i,1,5,o}$$

$$\frac{dX_{i,1,6,o}\left( t \right)}{dt}= {{{RR}_{o}^{ARTrecruit}\rho}_{1}X}_{i,1,3,o}+{{{RR}_{o}^{ARTrecruit}\rho}_{1}X}_{i,1,4,o}+{RR}_{o}^{ARTrecruit}{\rho_{1}X}_{i,1,5,o}+(1-{OAT}_{ODexit})\delta X_{i,1,6,o}-{{RR}_{o}^{ARTdropout}\psi_{1}X}_{i,1,6,o} -{(1-OAT}_{ODentry})\upsilon_{i}X_{i,1,6,o}-\left( \sigma_{6}+ ϛ_{i}+ \tau_{i}+ l+ {RR}_{o}^{ODint}\mu\right)X_{i,1,6,o}$$

Currently incarcerated

$$\frac{dX_{i,2,1,o}\left( t \right)}{dt}= {-\lambda}_{i}^{injprison}X_{i,2,1,o}- \lambda_{i}^{sexprison}X_{i,2,1,o}+ ϛ_{i}X_{i,1,1,o}+ \varepsilon_{s}X_{i,3,1,o}+ \varepsilon_{s}X_{i,4,1,o}-\left( \phi_{i}+ \tau_{i}+ l+{RR}_{o}^{ODint} \mu\right)X_{i,2,1,o}$$

$$\frac{dX_{i,2,2,o}\left( t \right)}{dt}= \lambda_{i}^{injprison}X_{i,2,1,o}+ \lambda_{i}^{sexprison}X_{i,2,2,o}+ ϛ_{i}X_{i,1,2,o}+ \varepsilon_{s}X_{i,3,2,o}+ \varepsilon_{s}X_{i,4,2,o}-\left( \sigma_{2}+\phi_{i}+ \tau_{i}+ l+ {RR}_{o}^{ODint}\mu\right)X_{i,2,2,o}$$

$$\frac{dX_{i,2,3,o}\left( t \right)}{dt}= {\sigma_{2}X}_{i,2,2,o}+ ϛ_{i}X_{i,1,3,o}+ \varepsilon_{s}X_{i,3,3,o}+ \varepsilon_{s}X_{i,4,3,o}-\left( \sigma_{3}+\phi_{i}+ \tau_{i}+ l+ {RR}_{o}^{ODint}\mu\right)X_{i,2,3,o}$$

$$\frac{dX_{i,2,4,o}\left( t \right)}{dt}= {\sigma_{3}X}_{i,2,3,o}+ ϛ_{i}X_{i,1,4,o}+ \varepsilon_{s}X_{i,3,4,o}+ \varepsilon_{s}X_{i,4,4,o}-\left( \sigma_{4}+\phi_{i}+ \tau_{i}+ l+ {RR}_{o}^{ODint}\mu\right)X_{i,2,4,o}$$

$$\frac{dX_{i,2,5,o}\left( t \right)}{dt}= {\sigma_{4}X}_{i,2,4,o}+ ϛ_{i}X_{i,1,5,o}+ \varepsilon_{s}X_{i,3,5,o}+ \varepsilon_{s}X_{i,4,5,o}-\left( \sigma_{5}+\phi_{i}+ \tau_{i}+ l+ {RR}_{o}^{ODint}\mu\right)X_{i,2,5,o}$$

$$\frac{dX_{i,2,6,o}\left( t \right)}{dt}= ϛ_{i}X_{i,1,6,o} {+{RR}_{o}^{recid}\varepsilon}_{i}X_{i,3,6,o}+ {+{RR}_{o}^{recid}\varepsilon}_{i}X_{i,4,6,o}- \phi_{i}X_{i,2,6,o}$$

Recently incarcerated (<6 months ago)

$\frac{dX_{i,3,1,o}\left( t \right)}{dt}= -{{RR}_{i}^{Rinc}\lambda}^{inj}X_{i,3,s,1,o}- \lambda^{sex}X_{i,3,s,1,o}+{(1-OD}_{prison}) \phi_{i}X_{i,2,s,1,o}+{(1-OAT}_{ODexit} )\delta X_{i,3,1,1,o}- (1-{OAT}_{ODentry})\upsilon X_{i,3,2,1,o}-\left( {\theta_{i}+{RR}_{o}^{recid}\varepsilon}_{i}+ \tau_{i}+ l+{RR}_{o}^{ODint} \mu\right)X_{i,3,s,1,o}$

$\frac{dX_{i,3,2,o}\left( t \right)}{dt}= {{RR}_{i}^{Rinc}\lambda}^{inj}X_{i,3,1,o}+ \lambda^{sex}X_{i,3,1,o}+{{(1-OD}_{prison}) \phi_{i}}_{i}X_{i,2,2,o}+ {(1-OAT}_{ODexit} )\delta X_{i,3,2,o}- (1-{OAT}_{ODentry})\upsilon X_{i,3,2,o}-\left( \sigma_{2}+{\theta_{i}+{RR}_{o}^{recid}\varepsilon}_{i}+ \tau_{i}+ l+ {RR}_{o}^{ODint}\mu\right)X_{i,3,2,o}$

$$\frac{dX_{i,3,3,o}\left( t \right)}{dt}= \sigma_{2}X_{i,3,2,o}+{{(1-OD}_{prison}) \phi}_{i}X_{i,2,3,o}+X_{i,4,3,o}+{RR}_{o}^{ARTdropout}{\psi_{3}X}_{i,3,6,o} + {(1-OAT}_{ODexit})\delta X_{i,3,3,o}-{{{RR}_{o}^{ARTrecruit}\rho}_{3}X}_{i,3,3,o}- (1-{OAT}_{ODentry})\upsilon X_{i,3,2,o}-\left( \sigma_{3}+{\theta_{i}+{RR}_{o}^{recid}\varepsilon}_{i}+ \tau_{i}+ l+ {RR}_{o}^{ODint}\mu\right)X_{i,3,3,o}$$

$$\frac{dX_{i,3,4,o}\left( t \right)}{dt}= \sigma_{3}X_{i,3,3,o}+{(1-OD}_{prison}) \phi_{i}X_{i,2,4,o} +(1-{OAT}_{ODexit})\delta X_{i,1,4,o}-{RR}_{o}^{ARTrecruit}{\rho_{3}X}_{i,3,4,o}- (1-{OAT}_{ODentry})\upsilon X_{i,3,4,o}-\left( \sigma_{4}+{\theta_{i}+{RR}_{o}^{recid}\varepsilon}_{i}+ \tau_{i}+ l+ {RR}_{o}^{ODint}\mu\right)X_{i,3,4,o}$$

$$\frac{dX_{i,3,5,o}\left( t \right)}{dt}= \sigma_{4}X_{i,3,4,o}+{{(1-OD}_{prison}) \phi}_{i}X_{i,2,5,o} +(1-{OAT}_{ODexit})\delta X_{i,3,5,o}-{{{RR}_{o}^{ARTrecruit}\rho}_{3}X}_{i,3,5,o}- (1-{OAT}_{ODentry})\upsilon X_{i,3,5,o}-\left( \sigma_{5}+{\theta_{i}+{RR}_{o}^{recid}\varepsilon}_{i}+ \tau_{i}+ l+ {RR}_{o}^{ODint}\mu\right)X_{i,3,5,o}$$

$$\frac{dX_{i,3,6,o}\left( t \right)}{dt}= {RR}_{o}^{ARTrecruit}{\rho_{3}X}_{i,3,3,o}+{{{RR}_{o}^{ARTrecruit}\rho}_{3}X}_{i,3,4,o}+{{{RR}_{o}^{ARTrecruit}\rho}_{3}X}_{i,3,5,o}-{{{RR}_{o}^{ARTdropout}\psi}_{3}X}_{i,3,6,o}+\phi_{i}X_{i,2,6,o} - (1-{OAT}_{ODentry})\upsilon X_{i,3,6,o}+(1-{OAT}_{ODexit}) \delta X_{i,1,6,o}-\left( \sigma_{6}+{\theta_{i}+{RR}_{o}^{recid}\varepsilon}_{i}+ \tau_{i}+ l+{RR}_{o}^{ODint} \mu\right)X_{i,3,6,o}$$

Not recently incarcerated (>6 months ago)

$$\frac{dX_{i,4,1,o}\left( t \right)}{dt}= \kappa_{i}\gamma_{i,r}\omega_{i}\left[ lN+\sum_{i} \tau_{i}N_{i} \right]-{RR}_{i}^{NRInc}{{RR}_{o}^{HIVint}\lambda}^{inj}X_{i,4,1,o}- \lambda^{sex}X_{i,4,1,o}- {(1-OAT}_{ODentry})\upsilon X_{i,4,1,o}+ {(1-OAT}_{ODexit})\delta X_{i,3,1,o}+ \theta_{i}X_{i,3,1,o}-\left( {RR}_{o}^{recid}\varepsilon_{i}+ \tau_{i}+ l+ {RR}_{o}^{ODint}\mu\right)X_{i,4,1,o}$$

$$\frac{dX_{i,4,2,o}\left( t \right)}{dt}= {RR}_{i}^{NRInc}{{RR}_{o}^{HIVint}\lambda}^{inj}X_{i,4,1,o}+ \lambda^{sex}X_{i,4,1,o}- {(1-OAT}_{ODentry})\upsilon X_{i,4,2,o}+ {(1-OAT}_{ODexit})\delta X_{i,3,2,o}+ \theta_{i}X_{i,3,2,o}-\left( \sigma_{2}+ {{RR}_{o}^{recid}\varepsilon}_{i}+ \tau_{i}+ l+ {RR}_{o}^{ODint}\mu\right)X_{i,4,2,o}$$

$$\frac{dX_{i,4,3,o}\left( t \right)}{dt}= \sigma_{2}X_{i,4,2,o}+(1-{OAT}_{ODexit})\delta X_{i,3,3,o}+{RR}_{o}^{ARTdropout}{\psi_{4}X}_{i,4,6,o}+ \theta_{i}X_{i,3,3,o}- {{RR}_{o}^{ARTrecruit}\rho_{4}X}_{i,4,3,o}-(1-{OAT}_{ODentry})\upsilon X_{i,4,3,o}-\left( \sigma_{3}+ {{RR}_{o}^{recid}\varepsilon}_{i}+ \tau_{i}+ l+ {RR}_{o}^{ODint}\mu\right)X_{i,4,3,o}$$

$$\frac{dX_{i,4,4,o}\left( t \right)}{dt}= \sigma_{3}X_{i,4,3,o}+(1-{OAT}_{ODexit})\delta X_{i,3,4,o}+ \theta_{i}X_{i,3,4,o}{{- {RR}_{o}^{ARTrecruit}\rho}_{4}X}_{i,4,4,o}-(1-{OAT}_{ODentry})\upsilon X_{i,4,4,o}-\left( \sigma_{4}+ {{RR}_{o}^{recid}\varepsilon}_{i}+ \tau_{i}+ l+ {RR}_{o}^{ODint}\mu\right)X_{i,4,4,o}$$

$$\frac{dX_{i,4,5,o}\left( t \right)}{dt}= \sigma_{4}X_{i,4,4,o}+ \theta_{i}X_{i,3,5,o}+{(1-OAT}_{ODexit}) \delta X_{i,4,5,o}-{{RR}_{o}^{ARTrecruit}\rho_{4}X}_{i,4,5,o}- {(1-OAT}_{ODentry})\upsilon X_{i,4,5,o}-\left( \sigma_{5}+ {RR}_{o}^{recid}\varepsilon_{i}+ \tau_{i}+ l+ {RR}_{o}^{ODint}\mu\right)X_{i,4,5,o}$$

$$\frac{dX_{i,4,6,o}\left( t \right)}{dt}= {{{RR}_{o}^{ARTrecruit}\rho}_{4}X}_{i,4,3,o}+{{{RR}_{o}^{ARTrecruit}\rho}_{4}X}_{i,4,4,o}+{RR}_{o}^{ARTrecruit}{\rho_{4}X}_{i,4,5,o}+ \theta_{i}X_{i,3,6,o}-{{{RR}_{o}^{ARTdropout}\psi}_{4}X}_{i,4,6,o}-(1-{OAT}_{ODentry})\upsilon X_{i,4,6,o}+ {(1-OAT}_{ODexit})\delta X_{i,4,6,o}-\left( \sigma_{6}+ {{RR}_{o}^{recid}\varepsilon}_{i}+ \tau_{i}+ l+{RR}_{o}^{ODint} \mu\right)X_{i,4,6,o}$$

κ_i_, γ_i,r_ and ω_s_ designate the distribution of individuals by sex i, by incarceration stage r for each sex i and by syringe

confiscation exposure s at entry, respectively. σ is the rate of progression from each disease stage to the next. l is the average mortality rate assumed to be equal among men and women while τi is the rate at which individuals stop injecting by sex. Ϛ_i_ and ε_i_ represent the primary incarceration rate among never incarcerated PWID and the re-incarceration rate by sex, respectively. φ_i_ represents the rate at which individuals exit prison, by sex and δ is the rate at which recently incarcerated individuals progress to the “non-recently incarcerated” stage. υ, is the rate at which men and women who inject drugs are recruited onto the intervention (either OAT or CAP) and δ is the rate at which they exit this state.

Modelling overdose

As the model was stratified by intervention (OAT or CAP) status, those currently receiving OAT had a reduced risk of overdose equal to μ (background overdose mortality rate) x RROD^OAT^. Conversely, individuals who were recruited to CAP, had an increased risk of overdose equal to μ x RROD^CAP^ We additionally incorporated an elevated risk of overdose in the first 4 weeks entering or leaving OAT based on data from systematic-reviews and meta-analyses[2]. Hence, we incorporate death for a small proportion of those entering OAT (OAT_ODentry_ ) , where OAT_ODentry_ = μ x (RROD^OATin^ – 1) x (4/52) . We multiply all of this by the duration at risk which is 4 weeks / 52 weeks per year. Similarly, when coming off the intervention, we incorporate death for a small proportion of those exiting OAT (OAT_ODexit_) where OAT_ODexit_ = μ x (RROD^OATout^ – 1) x (4/52). We used a similar approach when incorporating the risk of overdose within the first four weeks of prison compared to the risk among those released within the past five to twelve weeks [3]. We modelled this as OD_prison_ = μ x (RROD^prison^ – 1) x (4/52). The inflow of susceptible PWID was replaced by the number of PWID (N) who ceased from injecting or died due to background (non-HIV, non-overdose) causes.

**Force of Infection**

Outside of prison

$W_{p_{i}}= \frac{\beta_{2}\sum_{s,r=1,3,4} X_{i,r,s,2}+\beta_{3}\sum_{s,r=1,3,4} X_{i,r,s,3}+\beta_{4}\sum_{s,r=1,3,4} X_{i,r,s,4}+\beta_{5}\sum_{s,r=1,3,4} X_{i,r,s,5}+\beta_{6}\sum_{s,r=1,3,4} X_{i,r,s,6}}{\sum_{s,r=1,3,4} X_{i,r,s,h}}$

Where ART reduces parenteral transmission by a factor of RR^ARTinj^ as denoted below:

$$\beta_{6}={RR}^{ARTinj}* \beta_{3}$$

$\lambda_{i,j}^{inj}= C_{i}\omega_{i,j}{Wp}_{j}$

$\lambda_{i,j}^{inj}\left( t \right)= \sum_{j} \lambda_{i,j}^{inj}$

If on OAT or CAP, then ${RR}^{HIVint}*\lambda_{i,j}^{inj}\left( t \right)= \sum_{j} \lambda_{i,j}^{inj}$ where RR^HIVint^ is either the reduced risk of HIV transmission if on OAT or increased risk of syringe sharing if in CAP

In prison

${Wp}_{i}^{prison}= \frac{\beta_{2}\sum_{s,} X_{i,2,s,2}+\beta_{3}\sum_{s,} X_{i,2,s,3}+\beta_{4}\sum_{s,} X_{i,2,s,4}+\beta_{5}\sum_{s} X_{i,2,s,5}+\beta_{6}\sum_{s,} X_{i,2,s,6}}{\sum_{s,h} X_{i,2,s,h}}$

$\lambda_{i}^{inj,prison}\left( t \right)= C_{i}\omega_{i,i}{Wp}_{i}^{prison}$

Sexual force of infection


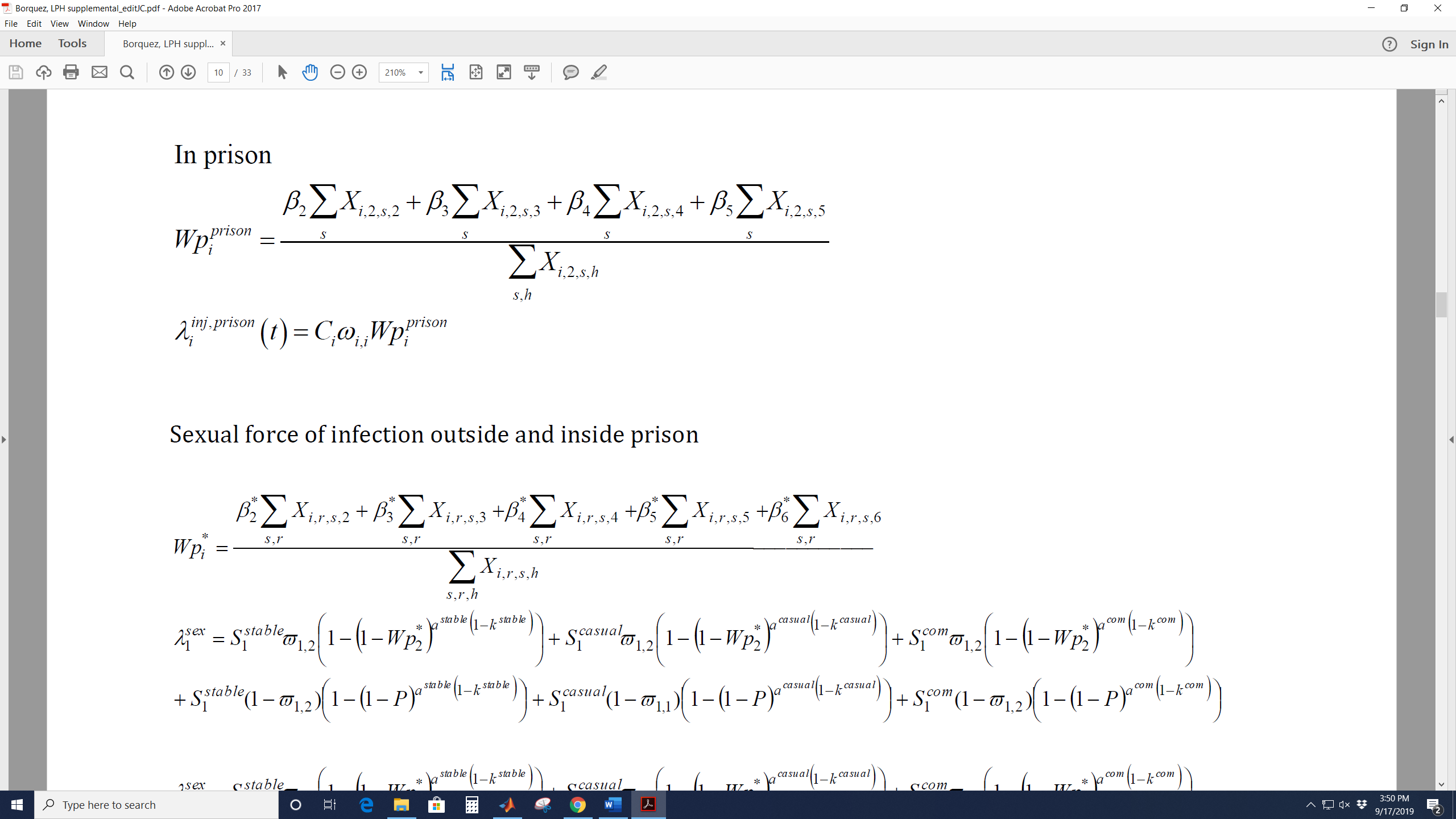


Where ART reduces sexual transmission by a factor of RR^ARTsex^ as denoted below:

$$\beta_{6}^{*}={RR}^{ARTsex}* \beta_{3}^{*}$$

$$\begin{matrix} \lambda_{1}^{sex}=S_{1}^{stable}\varpi_{1,2}\left(1-\left(1-Wp_{2}^{*}\right)^{a^{stable}\left(1-k^{stable}\right)}\right)+S_{1}^{casual}\varpi_{1,2}\left(1-\left(1-Wp_{2}^{*}\right)^{a^{casual}\left(1-k^{casual}\right)}\right)+S_{1}^{com}\varpi_{1,2}\left(1-\left(1-Wp_{2}^{*}\right)^{a^{com}\left(1-k^{com}\right)}\right) \\ +S_{1}^{stable}(1-\varpi_{1,2})\left(1-\left(1-P\right)^{a^{stable}\left(1-k^{stable}\right)}\right)+S_{1}^{casual}(1-\varpi_{1,1})\left(1-\left(1-P\right)^{a^{casual}\left(1-k^{casual}\right)}\right)+S_{1}^{com}(1-\varpi_{1,2})\left(1-\left(1-P\right)^{a^{com}\left(1-k^{com}\right)}\right) \\ \\ \lambda_{2}^{sex}=S_{2}^{stable}\varpi_{2,1}\left(1-\left(1-Wp_{1}^{*}\right)^{a^{stable}\left(1-k^{stable}\right)}\right)+S_{2}^{casual}\varpi_{2,1}\left(1-\left(1-Wp_{1}^{*}\right)^{a^{casual}\left(1-k^{casual}\right)}\right)+S_{2}^{com}\varpi_{2,1}\left(1-\left(1-Wp_{1}^{*}\right)^{a^{com}\left(1-k^{com}\right)}\right) \\ +S_{2}^{stable}(1-\varpi_{2,1})\left(1-\left(1-P\right)^{a^{stable}\left(1-k^{stable}\right)}\right)+S_{2}^{casual}(1-\varpi_{2,1})\left(1-\left(1-P\right)^{a^{casual}\left(1-k^{casual}\right)}\right)+S_{2}^{com}(1-\varpi_{2,1})\left(1-\left(1-P\right)^{a^{com}\left(1-k^{com}\right)}\right) \end{matrix}$$

Full details regarding the modelling equations can be found in the paper by Borquez et al [1]. Briefly, we modeled parenteral transmission in the community as a function of the number of receptive syringe sharing contacts (number of injections per month, the proportion who report any recent receptive sharing, the proportion who report receptive sharing at last injection among never incarcerated), the proportion of sharing contacts by sex, and the weighted HIV prevalence among syringe sharing partners by sex. Wp, accounts for the differential transmission probability by HIV stage, where those in the acute and pre-AIDS stages had higher viremia.

We assumed sexual mixing by sex between PWID to be exclusively heterosexual. Since a small proportion of PWID report having sex with other PWID (1·5% of men, 10% of women), in addition to the sexual transmission between PWID, an external force of infection was modeled to account for sexual transmission with non-PWID sexual partners. The prevalence of HIV among non-PWID sexual partners (0·5% to 1%) was based on estimates for Tijuana [4]. The rate of sexual transmission was treated as a function of the number of sexual partners, Si, sex acts per partner, a, frequency of condom use, k, with each of the different partner types (stable, casual and commercial) and of the HIV prevalence among sexual partners. A proportion of sexual partnerships happen between PWID, ϖi,j and the remaining occur with sexual partners who are not PWID. When estimating transmission from sexual partners who are PWID, the weighted HIV prevalence by sex, i Wp , which accounts for the differential sexual transmission probability by HIV stage, is used. When estimating transmission from sexual partners who are not PWID, the overall HIV prevalence P and the baseline sexual transmission probability $\beta_{3}^{*}$are used.

**Model calibration**

A full description of calibration procedures can be found in Borquez et al [1]. The model was calibrated first to prison demography using a simplified closed cohort sub-model of PWID incarceration (see Borquez et al [1]). This model was used to determine the primary incarceration rate (i.e. incarceration rate among PWID who had never been incarcerated) and the proportion ever incarcerated among PWID before injection initiation Based on the observed El Cuete IV baseline data, we calibrated this to the observed proportion of PWID ever incarcerated by duration of injection and by sex using Latin Hypercube Sampling (LHS) to sample 10,000 parameter sets and selecting fits which lay within the 95% confidence intervals of the data. A total of 56 parameter sets fit the observed data. These calibrated parameter sets were then used in the HIV transmission model.

We used LHS to sample from the distribution of parameters and the calibrated parameter sets from the prison demography described above. We used the beta and log normal distributions to sample values for proportions and relative risks respectively and used the uniform distribution to sample injecting and sexual behaviours to reflect wider uncertainty. The truncated normal distribution was used to sample incarceration parameters to reflect the data (point estimate and range). We ran 120,000 parameter sets to calibrate the model the proportion of PWID who were men in 2006, the total HIV prevalence in 2005, the HIV prevalence by sex in 2006, the HIV prevalence among ever incarcerated by sex in 2011, the relative HIV prevalence among never versus ever incarcerated by sex in 2011, HIV incidence by sex in 2013, the proportion of new infections attributable to sexual transmission in 2006 (estimated using an HIV/Syphilis co-infection model described in [1]), and ART coverage among HIV-infected PWID in 2012. We calculated the total log-likelihood as the sum log likelihoods for each of our calibration data points, and selected runs producing a log-likelihood above the 99^th^ percentile for the final analysis. In total, 201 runs were selected with most projections within the 95% uncertainty intervals (UI) of the data. Model calibration plots are shown in Figures S3-S4.

**References**

1. Borquez A, Beletsky L, Nosyk B, Strathdee SA, Madrazo A, Abramovitz D, et al. The effect of public health-oriented drug law reform on HIV incidence in people who inject drugs in Tijuana, Mexico: an epidemic modelling study. Lancet Public Health. 2018;3(9):e429-e37.

2. Sordo L, Barrio G, Bravo MJ, Indave BI, Degenhardt L, Wiessing L, et al. Mortality risk during and after opioid substitution treatment: systematic review and meta-analysis of cohort studies. bmj. 2017;357:j1550.

3. Merrall EL, Kariminia A, Binswanger IA, Hobbs MS, Farrell M, Marsden J, et al. Meta‐analysis of drug‐related deaths soon after release from prison. Addiction. 2010;105(9):1545-54.

4. Iñiguez-Stevens E, Brouwer KC, Hogg RS, Patterson TL, Lozada R, Magis-Rodriguez C, et al. Estimating the 2006 prevalence of HIV by gender and risk groups in Tijuana, Mexico. Gaceta medica de Mexico. 2009;145(3):189-95.
